# Parkinson’s Disease and Deep Brain Stimulation Have an Impact on My Life: A Multimodal Study on the Experiences of Patients and Family Caregivers

**DOI:** 10.3390/ijerph18189516

**Published:** 2021-09-09

**Authors:** Yolanda María Chacón Gámez, Florian Brugger, Nikola Biller-Andorno

**Affiliations:** 1 Institute of Medical Bioethics and History of Medicine, University of Zurich, Wintherthurerstrasse 30, 8006 Zurich, Switzerland; biller-andorno@ibme.uzh.ch; 2 Kantonsspital St. Gallen, Klinik für Neurologie, Haus 04 Rorsacher Strasse 95, 9007 St. Gallen, Switzerland; florian.brugger@kssg.ch

**Keywords:** deep brain stimulation, drawings, Parkinson’s disease, qualitative methods, patients’ and family caregivers’ narratives, personality, post-operative changes

## Abstract

Parkinson’s disease (PD) has a large impact on patients’ physical and mental health, which also greatly affects their family caregivers. Deep brain stimulation (DBS) has emerged as an effective treatment for PD, but different authors have expressed their concerns about the potential impact of DBS on personality and identity. Our study aims at better understanding how patients and family caregivers experience life with PD and DBS, the impact of both on their personal and social lives, and their perception of the changes that have occurred as a result of the disease and the treatment. Our study applies a multimodal approach by means of narrative semi-structured interviews and drawings. Seven principal themes have been identified: “everyone’s Parkinson’s is different”, “changing as a person during the disease”, “going through Parkinson’s together”, “DBS improved my life”, “I am treated with DBS but I have Parkinson’s still”, “DBS is not perfect”, and “being different after DBS”. PD is perceived as an unpredictable and heterogeneous disease that changes from person to person, as does the effect of DBS. While DBS side-effects may have an impact on patients’ personality, behavior, and self-perception, PD symptoms and drug side-effects also have a great impact on these aspects.

## 1. Introduction

Parkinson’s disease (PD) is one of the most common neurodegenerative disorders [1]. From 1990 to 2015, the number of people with PD doubled to over 6 million, and it is estimated to double to over 12 million by 2040 [2]. Although the incidence of PD increases with age, rising sharply around the age of 65, it does not affect only older individuals because cases of people with PD under 50 are not uncommon [2,3,4]. PD has become over the past two centuries one of the best-investigated disorders in neurology. It was first described in 1817 by James Parkinson when reporting six cases of ‘shaking palsy’, which was the term used at the time to refer to the phenomenology of disease [5]. In his monograph, he provided an already detailed description of this disorder including non-motor symptoms. A century later, Charcot provided a detailed description of this disorder including a description of non-motor symptoms associated with PD, which facilitated the diagnosis of PD worldwide [6]. Although PD is widely known for its motor and axial symptoms (e.g., tremor, slow movement, muscular rigidity, or postural instability) caused by the loss of striatal dopaminergic neurons, nonmotor symptoms are also very characteristic of PD and are due to the loss of non-dopaminergic neurons [7,8]. The non-motor manifestations of PD are very heterogeneous and can appear several years before the first motor symptoms [3,6]. They include fatigue, autonomic dysfunction (e.g., constipation, sexual dysfunction, or urinary retention), neuropsychiatric symptoms (e.g., anxiety, depression, dementia, or hallucinations), sensory symptoms (e.g., pain), and sleep disturbances (e.g., insomnia, REM-sleep behavior disorder or restless legs syndrome) [9,10]. Communication impairment is also common in PD and is associated with both motor and cognitive dysfunction [11,12]. All these symptoms have a large impact on PD patients’ physical and mental health, which can alter their individual and social identities and lead to a loss of autonomy and self-esteem, altered relationships, and social isolation [13]. Furthermore, it is a disease with a significant economic burden on society, payers, patients, and caregivers [14,15].

Motor and non-motor symptoms are managed through a variety of pharmacological treatments that include dopamine-based therapies for PD motor symptoms (e.g., levodopa, dopamine agonists) and nondopaminergic approaches (e.g., cholinesterase inhibitors, selective serotonin reuptake inhibitors) for nonmotor manifestations of PD. However, it has been known for a long time that under long-term levodopa therapy, patients often develop fluctuations in their motor performance (“wearing-off effect”) and dyskinesia [16]. Strategies to manage these complications include adding a dopamine agonist or inhibitors of levodopa-degrading enzymes such as catechol-O-methyltransferase or monoamine oxidase B inhibitors as adjuncts. However, these agents may also cause adverse side effects including a worsening of non-motor symptoms such as hallucinations, impulse control disorders, or gastrointestinal discomfort [17].

Device-aided therapies such as deep brain stimulation (DBS) can manage motor symptoms and improve the quality of life of patients with refractory tremor [18] or who suffer from intolerable side effects from oral treatment (e.g., dyskinesias from levodopa [19] or impulse control disorders from dopamine agonists [20]). Deep brain stimulation (DBS) emerged in the late 1960s as a possible therapeutic alternative for patients with chronic pain. In 1987, it was introduced as a therapy for PD by the French neurosurgeon Alim Benabid [21]. In 1997, the US Food and Drug Administration (FDA) approved the first DBS implant to treat tremor in PD and essential tremor [22]. In 2003, this approval was extended to treat other symptoms of PD. To date, this intervention is approved as a standard treatment by the European Union CE mark for PD, essential tremor, dystonia, obsessive compulsive disorder, and epilepsy [23], and several clinical studies are underway worldwide to extend the use of DBS to the treatment of other neurological and psychiatric diseases such as Tourette’s Syndrome [24] or treatment-resistant depression [25]. Although DBS has emerged over the past two decades as a treatment for both neurological and neuropsychiatric disorders, the main medical indication for DBS use is still represented by patients with PD [26].

DBS requires an invasive neurosurgical intervention that involves the insertion of electrodes deep into the brain, which are connected to a pulse generator placed in the chest region [27]. The device generates electrical pulses that stimulate a defined area of the brain. The most common stimulation targets for PD are the subthalamic nucleus (STN) or the globus pallidus internus (GPi) [28]. Therefore, this procedure works similarly to a pacemaker but for the brain [29]. In the bioethics literature, there is controversy about whether DBS has an impact on personality or identity. While different authors have expressed their concerns about the potential impact of DBS on personality and identity [30,31,32], others argue that there is not enough scientific evidence supporting this claim [33]. However, some quantitative studies show significant changes in personality and mood after applying DBS in PD [34,35]. We agree with other authors that pre-determined scales or standardized questionnaires may not capture the magnitude of all the changes in personality, identity, or self-perception that patients may face while being treated with DBS [36,37]. These changes could be due not only to DBS side effects but also to its interaction with PD progression and oral medication, as many patients continue to require medication, although usually in lower doses than before DBS intervention [7]. Furthermore, the changes that the patient undergoes are not only due to the disease or the treatment directly but also due to the process of adaptation that the patient goes through to become used to them [38]. Therefore, both from a clinical and from an ethics point of view, we consider it necessary to better understand qualitatively the experience of both having PD and being treated for it with DBS not only quantitatively but also qualitatively [39].

Although healthcare research is very much dominated by evidence based on quantitative research methods, qualitative approaches complement the medical doctors’ work in clinical routine because the approach applied by health professionals when seeing patient in day-to-day medical practice (e.g., when taking medical history) resembles methods used in qualitative research (e.g., interviews). Therefore, the results of qualitative studies are of great interest for neurologists and other clinicians as they serve to better understand the patients’ needs and tailor individualized treatment (e.g., choosing between DBS or medical pumps to treat motor fluctuations and dyskinesias). The available qualitative studies in the field have typically focused either on specific aspects of PD [40,41,42], the perception of DBS [38,43], or some side effects of DBS affecting patients’ personality or identity [44,45,46]. However, there are few studies with a comprehensive approach encompassing the patients’ experiences with both PD and DBS and deepening their perception of both [37,47]. To our knowledge, no qualitative studies have yet been carried out applying a multimodal approach [48].

Our study aims at collecting and analyzing a wide range of experiences of PD patients treated with DBS and their family caregivers. We applied a multimodal approach by means of narrative semi-structured interviews and drawings to better understand how they experience life with PD and DBS and the impact of both on their personal and social lives. We decided to include the point of view of family caregivers (FCs) because their experiences are a highly valuable source of knowledge for two reasons [49,50]. First, FCs are sometimes more sensitive to changes in the patients caused by either disease or therapy than patients themselves, and hence their experiences are of great value to complement the patient’s account [51]. Second, FCs provide daily care at home with PD and the different treatments for it, which modifies their personal routine and social life. Therefore, it is also worth to explore the impact of the disease and the treatment on the FCs’ personal lives.

This paper will present the experiences of nineteen patients and seventeen FCs with PD and DBS. We will first present our methodology and after the following themes will be covered: “everyone’s Parkinson’s is different”, “changing as a person during the disease”, “going through Parkinson’s together”, “DBS improved my life”, “I am treated with DBS but I still have Parkinson’s”, “DBS is not perfect”, and “being different after DBS”. Next, we will discuss our results and the strengths, limitations, and relevance for future research of our study. Finally, we will present our conclusions.

## 2. Materials and Methods

The set of data presented in this study is part of a bigger qualitative study conducted in Switzerland between 2018 and 2020, which explores a wide range of experiences of 44 patients and family caregivers with PD and two device-aided treatments: DBS and intrajejunal infusion of levodopa (known commercially as Duodopa^®^ pump). It applies a multimodal approach including narrative semi-structured interviews and drawings. This approach allowed a better exploration of the participants’ perception of PD and device-aided treatments incorporating both language-based and nonverbal communication. This study identified four groups of experiences reported by the patients and their family caregivers that can be classified in the following way: daily life and perception of PD and the effects of device-aided treatments, self-perception, social interaction and partnership/family dynamics, and experiences with different healthcare professionals including the receipt of PD diagnosis and the specific health needs of PD patients [52]. Due to the enormous amount of data obtained with this study and the different research questions we had, we decided to divide the results into three articles answering different questions [52,53]. Therefore, this article will focus on the first three categories mentioned above that are related to the experiences of PD patients treated with DBS and their FCs.

### 2.1. Researcher Characteristics and Reflexivity

The interviews have been conducted and analyzed by Researcher 1 (the first author) and Researcher 2. Researcher 1 (R1) is a PhD candidate working full time on the study from which this paper is derived. Researcher 2 (R2) is a postdoctoral researcher with extensive experience in qualitative methods. Both researchers are female and have been trained in DIPEx methodology [54]. There was no previous relationship between the researchers and the study participants. The participants did not know the professional characteristics of the researchers until the end of the interview.

### 2.2. Recruitment and Collaborations

The study has been conducted in collaboration with the Department of Neurology of different Swiss hospitals (Kantonspital St Gallen, Luzerner Kantonspital, and the University Hospital of Lausanne). We chose maximum variation purposeful sampling for the selection of participants to identify the individuals whose experiences were especially informative and would vary from each other as much as possible [55]. We therefore include participants from different parts of Switzerland with different symptoms and disease progression, different length of time with the disease and treatment, and different family environments and lifestyles. The participants were recruited through a combination of neurologists, PD nurses, and patients’ support groups belonging to the Swiss Parkinson’s association (Parkinson Schweiz).

For better dissemination of these results, they are part of the International Database of Patients Experiences research initiative (DIPEx International). This platform applies an established narrative method developed by the Health Experiences Research Group at the Nuffield Department of Primary Care at the University of Oxford [54]. The international DIPEx network comprises fourteen countries implementing their own national DIPEx platforms, which are based on qualitative studies. The aim of DIPEx is to present to a wide public (patients, family caregivers, health professionals, and students) a wide spectrum of diverse perspectives about different illnesses and health conditions [56,57]. Therefore, the Selected Material of the presented data in this paper will be uploaded to the Swiss DIPEx website in 2022 (www.dipex.ch, accessed on 4 May 2021) [53].

### 2.3. Study Population and Inclusion Criteria

A total of thirty-six Swiss people, including nineteen patients and seventeen FCs, participated in the study. In Table 1, the description of the patients (e.g., average age at interview, average age at diagnosis) can be found. One of the patients was treated simultaneously with DBS and the Duodopa^®^ pump (DP). Fourteen of the interviewed FCs were spouses of the patients, two of them were children (one son and one daughter), and one was the mother in law of one of the patients. Three participants participated alone. For two of them, the reason was that neither their spouses nor their children wanted to participate, and the other participant had no family in Switzerland. One of the FCs also participated alone because her husband was physically unable to participate in an interview. However, the patient was aware that his wife would participate in the study and agreed that she should share their story.

The inclusion criteria were as follows: (i) patients diagnosed with PD or relatives providing care to a PD patient and (ii) patients treated with DBS for at least six months or family caregivers providing care to PD patients treated with DBS for at least six months. The following exclusion criteria were applied: (i) lack of legal competency, (ii) people experiencing moderate or severe dementia or experiencing substance addiction at the moment of the interview, (iii) and lack of physical and psychological resilience to participate in an interview or difficulties interacting with an interviewer [53]. The inclusion and exclusion criteria were first evaluated and applied by the healthcare professionals that helped us to recruit our participants. They conducted a previous assessment of the participants who, according to their medical judgement, qualified for the study. Hereafter, R1 received a list of participants from the healthcare professionals and contacted the participants by telephone to make the appointment and ensure that the participants were able to maintain a rich conversation for a long time. To this aim, they were asked some questions about the disease and the treatment over the phone to ensure that they could share their experiences over different time frames (pre-disease, post-disease, and post DBS treatment).

### 2.4. Informed Consent Process

The participants were informed in great detail about the study before participating. On the day of the interview, they signed the informed consent form that allowed us to proceed with the interview and its subsequent analysis for our qualitative research. In this form, they also expressed their preference between being video- or audiotaped. After the interview, the participants received the interview transcripts for verification and the second informed consent form, through which they could accept or reject the use of the interview—either in video, audio, or text format—for the DIPEx website [54,56]. As a result, the number of participants in the DIPEx platform will be less than the number of participants in the study (*n* = 26/36 patients).

### 2.5. Data Collection

We applied a multimodal approach that includes the conduction of narrative semi-structured interviews and the collection of drawings. This multimodal methodology allows the collection of data about the experience with the disease and the treatment, incorporating both language-based and nonverbal communication to express their individual experiences and give them meaning [48,53,58]. We collected the data of the presented dataset, collecting drawings and conducting thirty-six interviews, of which thirty were conducted in 2019 and six took place in 2020. The German interviews were conducted by the first author (Researcher 1) and the French interviews by one of the members of our team (Researcher 2). The average duration of the interviews was 66 min for the patients and 53 min for the FCs. The duration of the interviews ranged from 27 min to 150 min. This duration does not include the time devoted to drawings. We conducted thirty interviews in German (with some parts in Swiss German) and six in French.

Thirty-two interviews were conducted at the participants’ homes, one was carried out in the nursing home where the patient lived, and three took place in our institute. They were conducted alone with the participant or in the company of the spouse, depending on the participant’s desire. Given that this study is part of the DIPEx databank, the narrative DIPEx method developed by the research group of the department of Public Health of the University of Oxford was applied to conduct the interviews [54,56]. Therefore, all interviews started with a narrative part to learn about the participants’ individual experiences, which was introduced with the following question: “Could you explain your experience with PD and DBS since the beginning?”. This question gave the participants great freedom to put their personal narrative into words [53].

**Figure A1:**
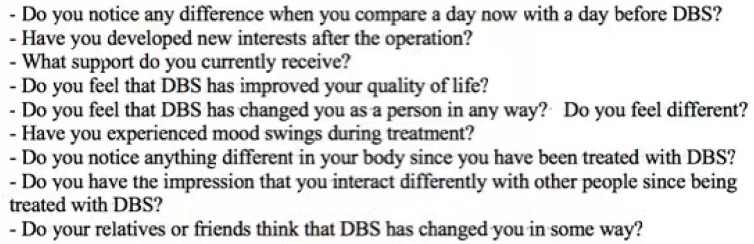
Extract of the interview guide.

This narrative part was further explored and complemented by semi-structured questions in the second part of the interview, which were structured in two sections. The first one was focused on the impact of PD on the participants’ lives (either as a patient or FC), including aspects such as the perception of PD, coping strategies, the experience with previous treatments (normally oral medication), and changes in self-perception, social life, and/or partnership/family dynamics due to PD. The second section comprehended questions focused on the participants’ perception of the DBS effects, their daily life with the treatment (either as patient or FC), the reasons to choose DBS as treatment, and the aspects that have improved or worsened with therapy. It also included changes in self-perception, social life, and/or partnership/family dynamics due to DBS. An extract of the interview guide for patients translated into English can be found in Appendix A Figure A1.

To offer additional insights to the researcher into the patients’ and FCs’ experiences with PD and DBS, we employed the drawings as a complementary qualitative method to the semi-structured interviews [53]. We collected drawings from 23 participants (14 patients and 9 FCs), and each of them made two drawings (one about their perception of the disease and another about the perception of DBS). One person only made the drawing about the perception of DBS. Therefore, we collected a total of 45 drawings. Thirteen participants opted not to draw due to physical incapability or the lack of a visual image of the disease or therapy. Although other authors asked their participants to draw after the interview [59], we decided to ask our participants to draw before conducting the interview to provide our participants with the opportunity to reflect on their own story before recounting it. We considered that this preliminary reflection would contribute to enriching the results obtained from the study [53]. Both patients and FCs were invited to draw and received a sketching pencil, a set of twelve color pencils, an eraser, and two pieces of paper. It was not required that the participants would know how to draw, as the only important thing was that what they drew had a meaning for them that they could explain. During the interview, the participants who drew were asked different questions about the drawings they made [53].

### 2.6. Data Analysis

We performed data analysis, applying a hybrid process of inductive and deductive thematic analysis, which required a continuous back and forth between data collection and data analysis [52,60,61]. To proceed with the analysis, the interviews were transcribed verbatim by our team of trained transcribers based on established rules [54]. After the participants’ verification, we fed the transcripts and the drawings into the qualitative data analysis software MAXQDA, which allowed us to perform multimodal analysis. After a first reading of each interview to familiarize with our data, we read every transcript several times while thematically coding the data using a coding scheme (coding tree). This coding tree was primarily created based on the interview guide, which served as a template [62]. For this reason, twelve initial codes were created from the questions included in the interview guide. These initial codes were the following: the meaning of drawings, getting the diagnosis, life with PD, the decision to undergo DBS, the DBS intervention, life with DBS, technical issues of DBS, relationships, wishes for the future, experiences with the medical team or in healthcare settings, suggestions or recommendations, and the reasons to participate in the study. This deductive approach was adopted in order to answer our research questions that sought to assess whether our findings were in line with the concerns expressed in the bioethics literature, which will be addressed in the discussion.

**Figure A2:**
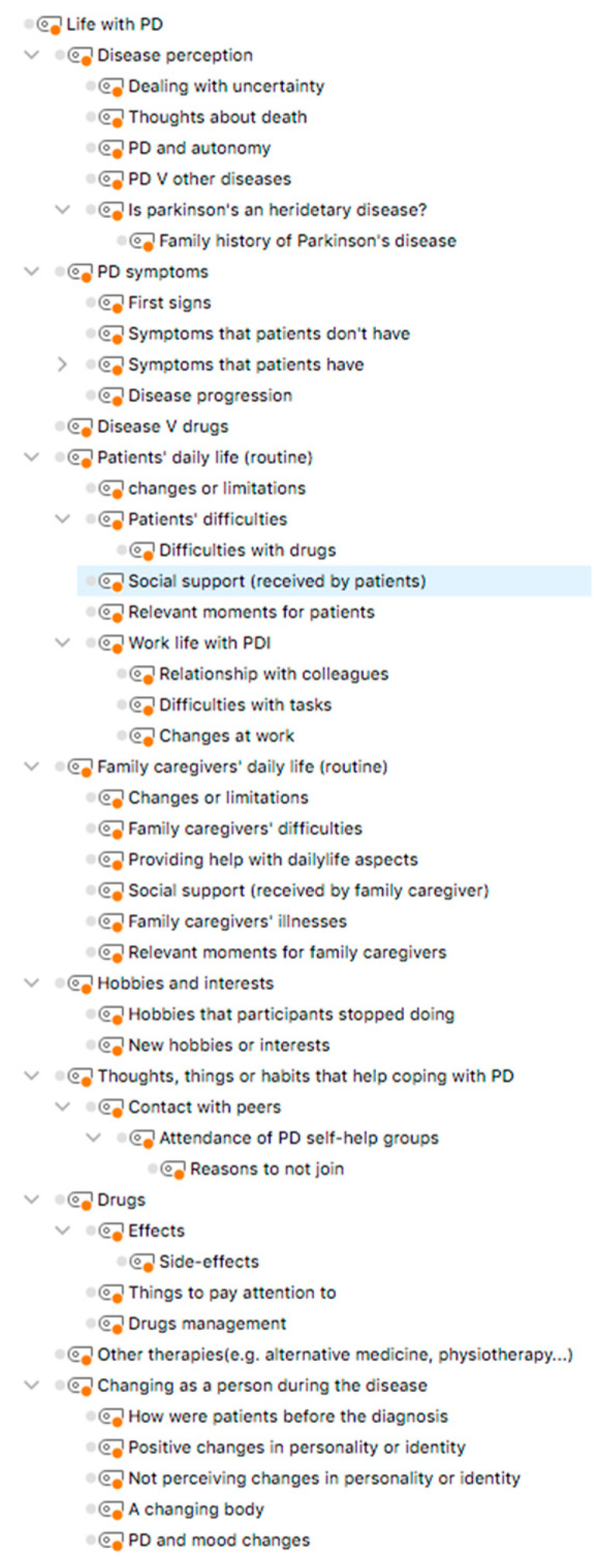
Extract of the coding three.

As we analyzed the interviews, we added more sub-codes within the initial codes created from the interview guide. In this way, the coding tree was continuously enriched with sub-codes that emerged from the interview transcripts and drawings (mix coding). Every time that we created a new sub-code, we reviewed all coded segments to ensure homogeneity within the entire data set [63]. This way, the concepts that we used to develop the final coding tree stemmed from the participants’ life experiences collected through semi-structured interviews and drawings, which were subsequently systematized, categorized, and analyzed following the coding tree [53,56]. Therefore, the analysis required a continuous back and forth between data collection and analysis to allow a constant comparison of the participants’ experiences and perspectives [64]. An extract of the final coding treatment can be found in Appendix A Figure A2.

Once we had analyzed all interviews and completed our coding tree, we sorted our codes into the descriptive themes presented in Table 2. We reviewed all themes to ensure that all extracts supported the theme and to avoid contradictions. We are aware that our themes allowed further abstraction, but we decided to stay with the descriptive themes that we present in this paper because they allowed us to more easily perform the next stage of the analysis in order to select the material for the Swiss DIPEx website. However, we do not rule out the possibility of a secondary study in the future to further develop themes that will allow us to further differentiate between the different nuances of our results.

The method for selection of relevant material for the DIPEx platforms is called the “one sheet of paper” (OSOP) method. It involves reading through each section of coded data for each topic and summarizing on a single sheet of paper the key points of all interviews in relation to the same topic [56,57]. The resulting text will constitute the summary of the different topics for the website. The website will be organized into the categories derived from the coding tree (e.g., PD symptoms, patients’ or caregivers’ difficulties with PD, work life with PD). All texts and video or audio clips on the website will be classified into these categories [53,54].

### 2.7. Quality Assurance of Data

Two interview guides were created, one for patients and one for FCs, which were tested before conducting the first interview to ensure that interview questions align with research questions. A total of 16% of the interviews were coded and analyzed by R1 and R2. They were first coded and analyzed by R2, and then by R1. Finally, both researchers reviewed both analyses, and after a thorough discussion, they decided on the final coding and analysis of this set of interviews. The rest of the interviews were coded and analyzed only by R1. The final coding tree was checked by another member of our team to ensure trustworthiness.

### 2.8. Ethical Concerns and Data Management

The study participants were not at risk of any physical harm and did not directly benefit from the study. We conducted this study in compliance with the current version of the Declaration of Helsinki, the ICH-GCP, and ISO EN 14155 (as far as applicable), as well as all national legal and regulatory requirements. The study has been reviewed by the ethics review committee of the Canton of Zurich, which considered that it did not fall under the Swiss Law on Human Subjects Research. Therefore, after consultation with the national working group of Swiss ethics review committees, the committee issued a nation-wide waiver (BASEC-Nr. req-2018-00050).

We handled all data confidentially, and only persons who were directly involved in the data collection, transcription, or data analysis had access to them. The data were stored in a server provided by our institute, and we performed data anonymization, saving the data of each participant by giving them a code (e.g., P1, FC1…). We also deleted from the transcripts any information that could lead to the personal identification of participants [53]. Only R1 has access to the document with the identification of the participants, which is encrypted for added privacy protection. In order to present our results in this paper, we translated the selected quotations of the interviews that were not conducted in English using the DeepL Pro Translator. The translated data were not stored by DeepL, and all translations were compliant with our data protection regulations. The final translations were verified by a native speaker to ensure accurate translation of the original quotes.

## 3. Results

The study revealed seven overarching descriptive themes, which contain different subthemes. These themes can be found in Table 2.

### 3.1. Everyone’s Parkinson’s Is Different

PD was considered by virtually all the participants to be a disease that presents itself in a vastly different way to each person and to which everyone reacts differently.

#### 3.1.1. Different Symptoms and Disease Progression

The symptoms experienced by the participants were very diverse and varied considerably from patient to patient. The most frequently mentioned symptoms were tremor, uncontrolled movements (dyskinesia), weight loss, fatigue, decreased facial expression, freezing of movement, sleeping disturbances, becoming slower, and having difficulties when walking (e.g., taking small steps) or talking (e.g., speaking too softly or not clearly). Many of the patients who experienced stiffness or walking difficulties did not experience tremors. Weight loss occurred more frequently in people who experienced dyskinesia. Other symptoms, reported by fewer people, were dizziness, shoulder pain, urinary incontinence, swallowing problems, and memory problems. Non-medication-related psychiatric symptoms and mood swings like touchiness, irritability, aggressivity, hallucinations, depressive episodes, and substance addiction were also reported.

“*I saw sand flowing from anywhere but in a closed circuit. It never stops (…) or I saw a black dog under the table at the hospital. There, I had a concentration of hallucinations from everyone. The doctors who came to see me at first that I was having these hallucinations, they had spaghetti hanging out*”.

(P44)

Suffering from psychological or psychiatric manifestations of PD were pointed out as a concern that patients have:

“*Many people with Parkinson’s disease say that if they have psychological problems from the disease, either from or with or because of the disease, it is much worse than if they tremble and cannot walk well. And I think that too*”.

(P27)

One FC described how changes in mood can trigger PD motor symptoms:

“*About 15 years ago, she made a mistake with our boat and hit a buoy at high speed. Then the water police came (…) She was trembling all over her body and I have never seen her like this. That frightened me very much and since then, it happens whenever she gets upset (…) When she is tense, she has such uncontrolled movements. I first noticed in 2004 that when she is very excited or scared or something, she trembles*”.

(FC13)

Not only do PD symptoms differ from patient to patient, but the disease progression also varies for each of them. Several participants explained that the progression of PD was very slow during the first decade. During this time, the disease was well controlled with low medication and, in one case, without taking any medication during the first ten years. All of them were grateful that they could continue doing things that were important to them (e.g., working or snowboarding) for a decade without experiencing many limitations. This period was referred to as “the honeymoon” by some participants. However, at a certain point, it came to an end, and the disease then progressed more rapidly, which required progressive increasing of medication over time. The worsening of the disease manifested in two different ways. Some patients experienced aggravation of symptoms that they already had (e.g., more fatigue or dyskinesias), while others noticed new symptoms (e.g., quieter voice, problems when walking). This progression brought new limitations on the patients that they did not have previously, such as being unable to continue working.

“*In the beginning, I experienced the honeymoon, it went great, and the more the disease progressed, the more the uncontrolled movement I had. (…) I lost 10 kilos in half a year because I couldn’t sit quietly anymore*”.

(P9)

Other patients experienced a much a faster PD progression from the beginning. The manifestation of new symptoms was very difficult for the FCs to cope with:

“*This has changed a lot, the disease. That is quite clear. Then, as you can see with the disease, where dyskinesia became more and more pronounced, comes the physiognomy change. I no longer knew my own wife by her face. It was so bad, the disfigurement that was caused by this illness that affects not only the movements but also the face*”.

(FC22)

#### 3.1.2. Different Perception of the Disease

Since every participant, both patients and FCs, was affected by PD in a different way, their perceptions of the disease varied greatly as well. Some participants have the impression that PD is not a fatal disease like cancer because it does not shorten life expectancy. PD was described by several participants as a disease for which physical activity was essential to relieve its symptoms. As sporting activity was one of the most important things in life for one of the patients, they explained that another disease would have been worse for them than PD. Other participants considered that many things are still unknown about PD. For many of the participants, PD marked a before and after point in their lives:

“*This is the life before and after the disease and then you learn to live with it. It is true that I have done a lot of research to find out where we were heading to (…) We were told that there are as many symptoms as there are sick people. And this is how it is*”.

(P41)

In this sense, one of the participants defined PD as a family of diseases that affect every person differently rather than a just one disease:

“*I had the impression that Parkinson’s itself does not exist and that it is a sum of factors that are individual. Therefore, they are really not understood, and they are mentioned below the name of Parkinson’s (…) If there would be a better understanding of the whole mechanisms, one could finally say that there are many versions of Parkinson’s, which are entirely related*”.

(P1)

P1 also described PD as a disease that leads to the patient being automated and no longer in control of himself. Some patients depicted PD as a disease that evolves and worsens over time. Therefore, one needs to enjoy every day but also learn to cope with living with the disease every day.

“*I can just say that my attitude to life is like this, I fight as long as I can, but it’s just getting harder and harder. It’s really getting more and more difficult. (…) This disease is also very interesting in terms of what you forget when it changes again afterwards*”.

(P12)

**Figure 1:**
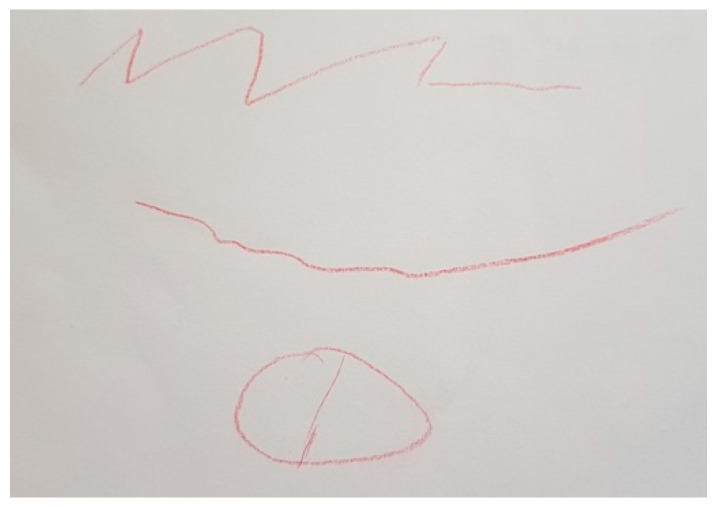
P28’s perception of PD.

Some participants drawings reflected how it makes them feel to have PD or to have a loved one with PD. In Figure 1, we observe how P28 perceived PD as a succession of ups and downs that made him feel better or worse. To the patient the eye represents that both himself and the people around him can perceive the fluctuation of symptoms.

**Figure 2:**
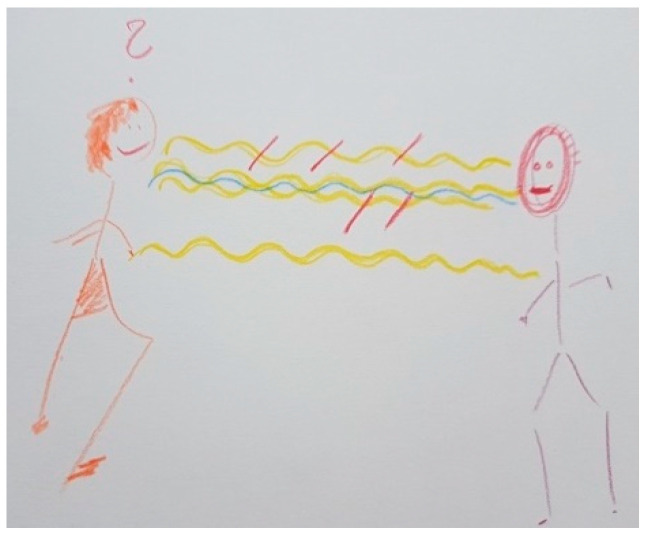
FC6’s perception of PD.

Figure 2 shows how the facial stiffness characteristic of PD makes relationships and communication difficult. The yellow waves symbolize the psychological, empathetic, and linguistic connection with her husband. The short vertical red lines reflect the loss of connection with her husband and the interruptions in their communication as a consequence of the disease. The blue wave represents her effort to maintain communication with her husband.

“*What I drew is what concerns me the most and touches me negatively, which is the effect as if he would have a mask on his face (…). I always have to think when I talk to him or when I get in touch with him whether there’s something going on. He just looks glassy-eyed. It’s not that there’s something wrong or that something has happened (…) It is somehow difficult for him to express that he is there for the other person in an empathic way. And the connection is somehow interrupted. But maybe that has to do with me too. I react very personally*”.

(FC6)

**Figure 3:**
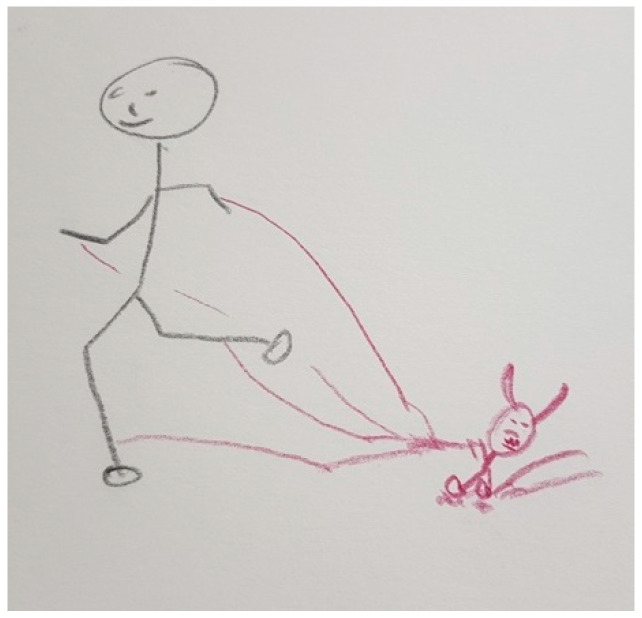
P5’s perception of PD.

**Figure 4:**
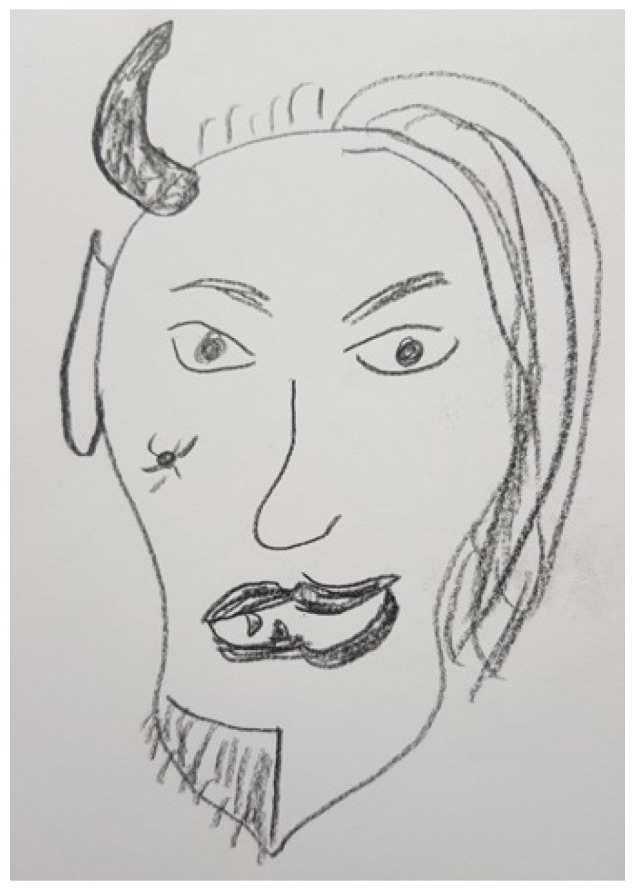
FC29’s perception of PD.

**Figure 5:**
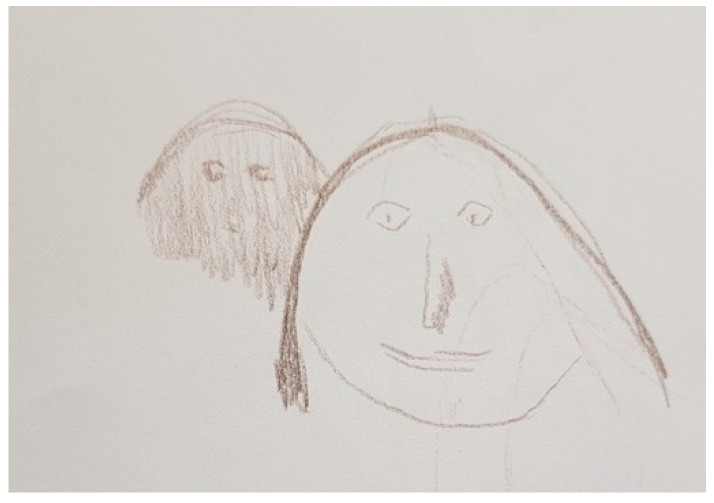
P35’s perception of PD.

**Figure 6:**
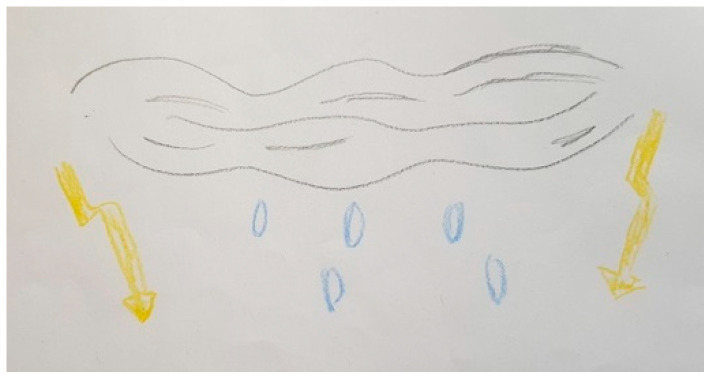
P40’s perception of PD.

**Figure 7:**
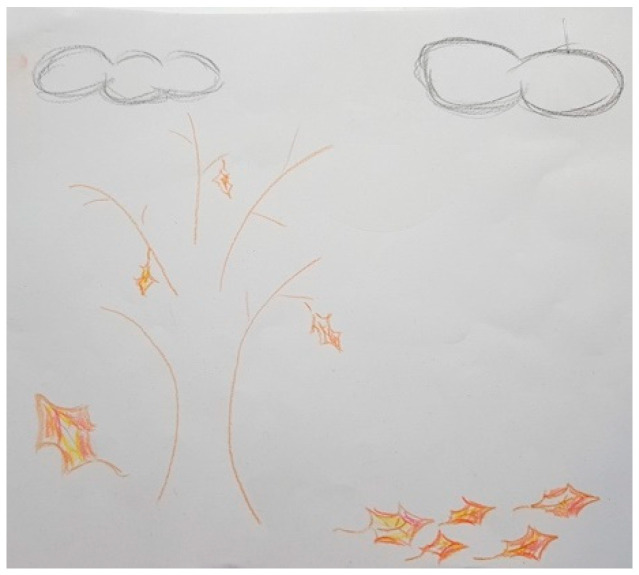
FC41’s perception of PD.

Several participants, both patients and FCs, represented PD in their drawings with different figures that show the impact of the disease on their lives. These figures are very varied and range from the representation of PD as a creature that is always there, like in Figure 3, Figure 4 and Figure 5, to describing PD by analogy to the weather or the seasons of the year, like in Figure 6 and Figure 7.

“*That’s me. I have several ropes attached to my legs and arms and a little demon that holds me back. Then, my mobility is limited*”.

(P5)

“*I think the disease itself is the devil on one side and the beautiful fairy on the other side. It is entirely day-dependent, time-dependent. Sometimes you could almost despair about the disease and other times, everything it’s quite normal and you can say to yourself that everyday life is actually quite normal. And then in the next half hour nothing works at all. What is also the problem is that we have to prepare every appointment very carefully*”.

(FC29)

“*This is me, actually a satisfied person. And he has a shadow. And he doesn’t have an angry face, but he’s looking to see how I am (…) I’m actually happy when he stays behind me, when he doesn’t come in front of me (…) Whether I like it or not, he’s always there. I can’t turn him off*”.

(P35)

“*It’s to explain the bad weather, the worst of the bad weather, for me. I was very active. I was really someone who was moving and all of sudden I had to stop because it was no longer possible, and my body wasn’t following*”.

(P40)

In Figure 7, we can observe how PD is perceived as a before and after in the lives of both patients and FCs.

“*It [PD] is autumn. Autumn. The leaves falling is not the end of life, but it’s the end of a life. It’s the end of the life before illness. So, it’s a time to mourn*”.

(FC41)

**Figure 8:**
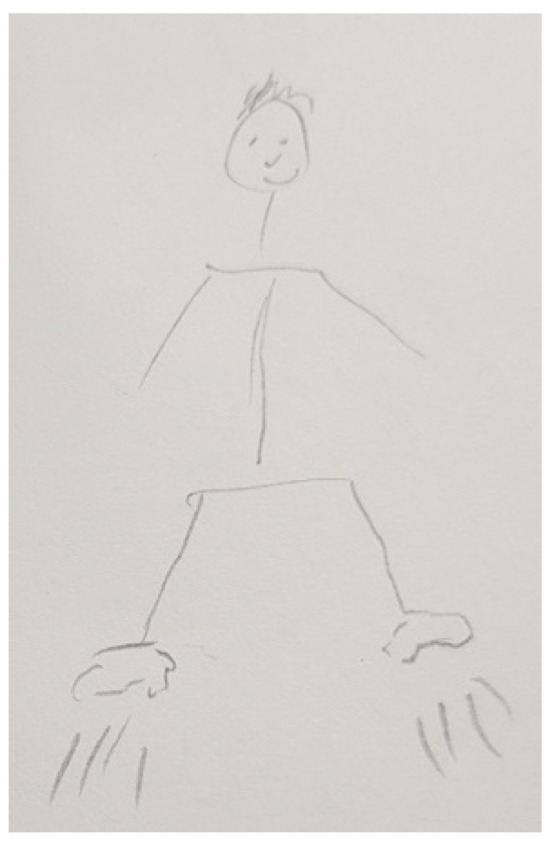
P21’s perception of PD.

**Figure 9:**
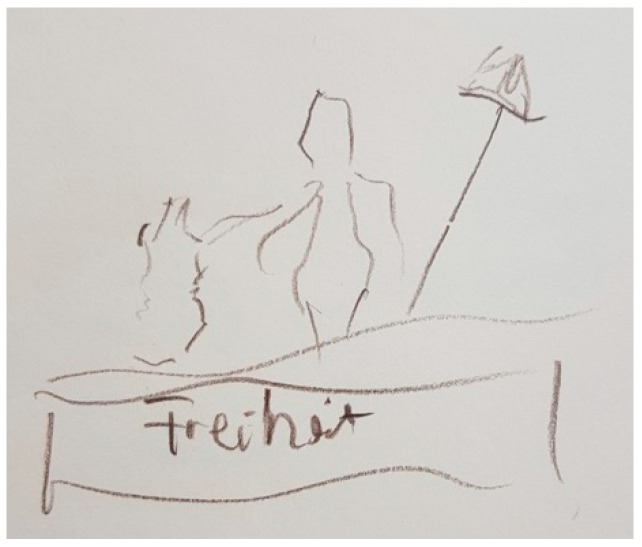
P8’s perception of PD. The word “Freiheit” in German means freedom.

Some patients visually perceive PD as the lack of movement or freedom (e.g., Figure 8 and Figure 9), rather than as concrete and defined figures, whose symbolism explains the impact on their life (e.g., Figure 3, Figure 4, Figure 5, Figure 6 and Figure 7).

“*There’s glue on your feet. You want to go somewhere, and you can’t. In your head you are already there, but your body cannot go there because your feet are sticky. The dopamine is missing*”.

(P21)

“*This freedom, I don’t have it anymore, because I have Parkinson’s disease and I’ve lost my freedom. I find that very bad. I find it so sad (…) Now I simply have a good life, a good care, but I do not have freedom anymore*”.

(P8)

This feeling of loss of freedom has also been described by some FCs:

“*Parkinson’s is like a cage for the person. The person is like in a cage for me and I can’t get into this cage*”.

(FC6)

#### 3.1.3. Different Reaction to Drugs

Most participants had good experiences with the medication until it had to be increased. However, a few explained that the medication never helped to control the disease symptoms. While some participants found it difficult to distinguish between disease symptoms and side effects, others considered that certain effects were side effects of the medication and not PD symptoms. This impression is due to the disappearance of symptoms once the dosage of the medication was decreased at the start of treatment with DBS. A wide variety of side effects were reported, such as diarrhea, stomach pain, dry mouth, lost sense of taste, increased libido, various addictions (e.g., to sex or compulsive shopping), hyperactivity, aggressivity, and depression.

“*Our friends also noticed that he was so uninhibited and always restless. He always had to be stimulated and always had to listen to music. He also bought a lot of things like cars. He had a different behavior*”.

(FC2)

The experience of hallucinations was the most mentioned side effect. In some cases, hallucinations appeared to or became more frequent after increasing the medication. The side effects of medication also have a direct impact on the FCs:

“*The increased libido is a problem after all. People don’t talk about it so much, but it’s a side effect of the medication and it’s very demanding for the partner*”.

(P1)

FC2 described her husband’s difficulties in controlling his impulses due to the medication he received before starting DBS treatment. She explained that this side effect was very challenging for family life because they had very young children at that time:

“*For example, when we were at the table with the children, he ate a lot and very quickly, and then stood up and went to his computer. And that’s difficult with children, when you try to educate the children and say: wait until everyone has finished and then you can get up and leave the table. And he, he had no concept of parenting anymore*”.

(FC2)

#### 3.1.4. Different Difficulties and Changes That Led to Different Daily Routines

PD symptoms are limiting and lead to changes in life daily routine. For example, several of them had to change their routine and concentrate all their tasks and activities during the day because in the evening they had no energy. The pace of doing things and the perception of time also changes during the disease, which changes daily routines for patients and FCs:

“*People with Parkinson’s, they lose their sense of reality over time. For example, when she cooks something, it goes in slow motion. When I say: ‘can’t be done faster?’. She says: ‘yes, I do work fast’. Or also with movements, when people with Parkinson’s walk, it’s centimeter by centimeter and when you ask them about it and say: ‘why don’t you take bigger steps? It’s better’, she says: ‘yes, I do take big steps’. It’s as if reality slowly disappears*”.

(FC29)

Some patients also experienced difficulties such as being less productive than before, performing daily tasks much slower, being less flexible, or not being able to do certain activities such as traveling or driving. Most of the patients who were still working at the time of diagnosis reported some difficulties at work that caused them great stress (e.g., difficulties while typing on a computer, speaking in public, staying focused, or performing physically demanding work). Due to these difficulties, many of them reduced their working time, delegated some of their tasks to others, or stopped working.

Other difficulties in daily life expressed by some participants were the need to plan every little thing well in advance, the impossibility to stop thinking about the disease, or the loss of autonomy by needing the help of others in daily activities:

“*I feel sometimes pressured because I make an appointment for her somewhere and calculate how long we need to get her ready and to be there. And then when we leave, it can happen that nothing works until we get to the station because it takes us a quarter of an hour to walk ten meters and then the train bye*”.

(FC29)

“*One loses independence and of course is somewhat limited. You’re glad to have someone by your side to help you up, for example, on the edge of the bed to get up*”.

(P9)

This dependence of some patients led to changes in the routine of FCs to be more available to their spouses. While some patients did not require much help from their spouses in daily life, in other cases, the FCs needed to take care of all the household chores or assist patients with personal hygiene. Both patients and FCs described feeling as if daily routine was marked by the disease. Another difficulty experienced by the participants was the necessity of preparing for the future changes and the uncertainty that PD entails, which is sometimes more difficult for FCs than for the patients themselves:

“*What comes next? How much should I work? Should I take this job or better this one? (…) How will it go financially and how will it go later? How many years will it take before he needs care? What will I do then? Can I care for someone at all? I’m not the caring type of person. I noticed that a long time ago. Then I thought, oh my God, how do I do that then? It was always like a sword of Damocles hanging over you and you have to think, what’s next?*”

(FC6)

#### 3.1.5. Different Impact on Familial and Couple Relationships and on Social Life

While some participants did not observe changes in their lives as a couple, others reported large changes in their relationships:

“*Something changed, I don’t know (…) Over time he became more of a patient and I became more of a caregiver, but I wouldn’t say from the beginning. It was over time, when more and more symptoms appeared (…) The illness has a strong influence on communication, because he often speaks very softly, because he speaks unclearly. Now, in the last few months, he is increasingly unable to express himself so well. The words don’t come out or he no longer knows what he wants to say, and his range of interests is simply more limited*”.

(FC17)

Many of our participants needed to adapt to great changes in their couple life including the impossibility of sharing their hobbies (e.g., travelling, skiing, taking long walks), having sex, or even sleeping together due to sleep disturbances caused by the patients’ PD. A few participants described having gone through marital crises as the disease progressed. A couple of patients reported lack of support from their partners during the illness due to the burden it places on FCs:

“*She does not want to talk about it* [PD] *(…) My wife is not understanding as others can be. It’s always like that, an illness, it always affects both the relatives and the affected persons themselves* (P7) *(…) Would you say that your relationship with your wife changed after the diagnosis?* (Researcher 1). “*Yes, yes. I think it has. Yes, yes. She can’t help like that either, can she? (…) There are some people who really still have complaints and I also have complaints, but those who can no longer walk or are really old people. And with such problems my wife simply has problems. She doesn’t want to see them at all*”.

(P7)

One of the patients explained that his second wife asked him for a divorce after finding out about the diagnosis.

“*In our wedding day, she told me that you were limping (…) And then I went to the doctor, first to the general doctor and then to the neurologist. The second wife, she asked for divorce. I actually understood that, because she had already known my mother and seen how it is (…) And my second wife couldn’t stand that*”.

(P8)

One FC described the relationship between her son and daughter-in-law deteriorated because he lost patience.

“*It’s just that for many couples it’s a big challenge. I see many who are trembling, and it’s easy, it wouldn’t work anymore if the partner didn’t have more patience. It’s tragic to see how that hurts you. There are so many different people. There are people who deal with it better and others who deal with it worse. You know, my son is washed up with it because he’s always had Parkinson’s around him. He experienced the grandfather yes. He came from school, he had to find the grandfather somewhere, he had to put him up again and bring him into the house. Then he experienced his father for 30 years. And now his partner and that is a lot for him at the moment and I think that often he just doesn’t have the energy anymore (…) She was very unwell before the operation and I just felt she had a nervous breakdown (…) I just notice that when she gets stressed, it comes through, and she needs another day* [to recover]. *And my son is allergic to it. He just almost can’t stand it. He didn’t want to be there today”* (FC24). *“Can I ask why?”* (R1). *“Something kind of broke”* (FC24). *“In the relationship you mean?”* (R1). *“Unfortunately. That hurts me a lot*”.

(FC24)

PD also had an impact on the family life of several patients and their relationships with their children. In cases where children were very young, some patients tried to avoid mentioning the disease in front of them, but the children still sensed the disease and were affected by it.

“*At one point he was quite affected, and I asked him the question. I said, but what’s wrong? Then he looked to me in the eye and said: are you going to die daddy? I said no, but no, but no! It had nothing to do with it. In fact we hadn’t explained it to him because we thought we were protecting him and then we realized that he was still worried about it*”.

(P40)

In many cases, adult children were a support for their parents with the disease. However, in other cases, the illness was a rarely discussed topic between the patients with their adult children. One of the participants described the deterioration of the relationship with their children:

“*It’s quite difficult, it depends. My wife doesn’t have a problem, but she understands everything. She has given herself body and soul for me. But the children are not the same at all. It has changed (…) let’s just say that they don’t understand so much that you’re sick*”.

(P44)

In relation to the impact of the disease on social life, some participants did not notice any changes in their friendships as a consequence of PD. While some of them consider that there were not changes because they did not talk about the disease with their friends, others thought that their friendships remained unchanged because they had talked openly about their illness from the very beginning. However, a few reported losing some friends due to the disease. Some also explained that due to the disease, they no longer felt comfortable organizing events, going out, or having too many people to visit, which greatly reduced their social life.

“*I didn’t want to show others this image of me, this image of the disease in fact”* (P40). *“So you didn’t go out anymore?”* (R2). *“Very little (…) I have the impression that some people think that we are not the same because we are sick. I think there are many who think that because maybe I’m a little slower, I have more difficulty in talking, that inside we’re not the same, whereas inside, when I think, I think very quickly*”.

(P40)

#### 3.1.6. Different Personal Coping Strategies

Most of our participants considered PD to be a disease that requires being addressed proactively, because they needed to do something to cope with it and make it more bearable. However, the same coping strategies did not work for all of them. For instance, while for some of them, looking for information about PD was a way of coping with the disease, others preferred not to know much about the illness to avoid feeling drowned by the situation:

“*In fact, I preferred* not *to know anything (…) I told myself that if I didn’t know anything, I wouldn’t have symptoms that could happen later*”.

(P40)

Coping strategies for PD described by the patients include practicing different sports or physical activities such as coordination training, walking, tai chi, snowboarding, dancing, or kickboxing; other activities such as starting a new hobby (e.g., doing a cooking course); or focusing on the family and specially playing with their grandchildren. Other helpful habits mentioned by the patients for coping mentally with PD were paying attention to diet or staying positive by valuing the small things of everyday life such as enjoying a sunny day or the forest colors. Other strategies to help patients cope with the physical symptoms of the disease were receiving daily leg massages in a massage chair, daily recording of the symptoms to keep track of them, reading aloud to train the voice, or resting the day before a social event to avoid being too tired. Strategies to help the patient walk included counting the steps or following someone while walking. The habits that help each patient with different aspects of the disease are very varied, but there is a consensus on the ideas that physical activity or being distracted contribute to the patient experiencing fewer symptoms while carrying out an activity. FCs also described several activities that helped them to cope better with their loved ones’ illness such as praying, reading books with characters with whom they can identify, or doing things alone to clear their mind. Having time for themselves doing activities they enjoy such as sports or socializing was also seen as a way of coping with the illness of their loved ones.

The contact with other people who have PD or who have family members with PD was mentioned as a support in coping with the disease for both patients and FCs. Many of the participants attended self-help groups specifically for patients with PD treated with DBS or for their relatives:

“*That is also the purpose of this self-help group, because people meet there who know what it means to have experienced this operation, but also what it means to have survived it. And what it means when other people think you are healthy again*”.

(P12)

Visiting a self-help group was an opportunity for the FCs to look after themselves by discussing their experiences and how they deal with the disease as FCs:

“*In my opinion, too much is said about the sick person in our self-help group and not about oneself in the relatives’ group. So, I always bring that up there (…) I ask: ‘how are you doing’ and now they are starting to talk a bit more about themselves. (…) It helps to talk to people who have similar experiences. So, it’s also like not being alone. Talking about it with other people is always a bit difficult if they judge how it should be (…) But with those who have the same experiences, you only say I do it this way or I do it differently. And, um, yes, you also have to look after yourself*”.

(FC17)

However, some patients and FCs expressed that speaking about the disease or seeing people in the same situation as them would have not helped. Instead, some FCs, who did not want to join self-help groups, talked about the disease with relatives, friends, or acquaintances as a way of coping with their loved ones’ disease. Sometimes, this was contrary to the individual patient’s way of coping with PD:

“*He had the impression that we were seeing something* [PD symptoms], *when it wasn’t at all (…) But he could see that, and he had the impression that everyone was paying attention to it. So, he didn’t want to talk about it too much, and I was the opposite. For me, to talk about it was a way of trivializing this illness. It was like saying I have the flu, yes, he has Parkinson’s, and then there you go. Then I talked a lot about it. It’s true, I pushed him to do it because it’s not keeping things inside that’s going to help*”.

(FC41)

### 3.2. Going through PD Together

As previously mentioned, FCs provide support in many aspects of daily life, such as help with getting out of bed, help with personal hygiene, accompanying patients to medical appointments, or doing activities with the PD patient to stimulate their memory or their motor skills:

“*I do everything for my partner. Doctor talks, everything, and I’m there and I want to know what’s being done and yes. That’s everything for me. And that’s why, we manage, we do everything. It works*”.

(FC14)

Although some patients were not supported by their spouses with the disease, most patients described the support and understanding provided by their spouses as invaluable for dealing with the disease:

“*I think I wouldn’t have made it without her*”

(P44)

**Figure 10:**
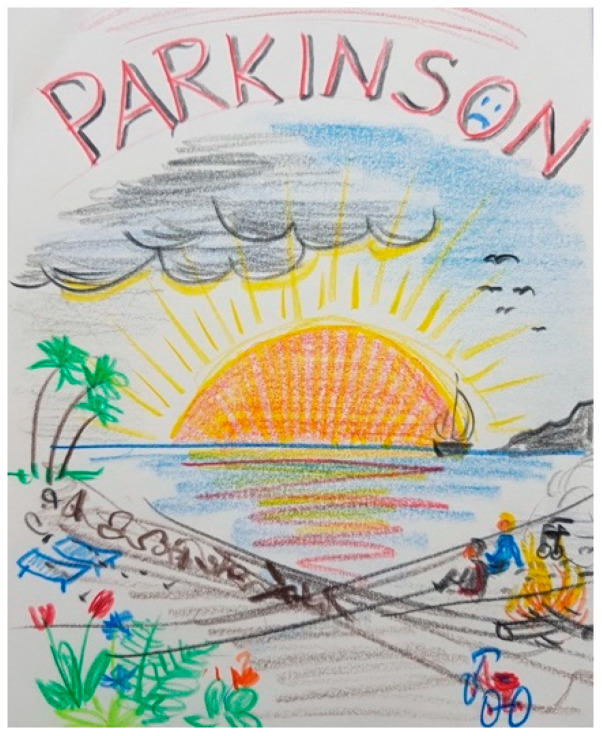
FC26’s perception of PD as a joint journey for the couple.

PD was a shared experience for several participants, as shown in Figure 10. This figure also portrays how some couples tried to enjoy as much as possible together despite one of them having PD.

“*It is a double-edged sword. Dark clouds and clear bright sky (…) I have painted a small campfire here, and my wife’s tricycle, with the walker standing next to it. We live with these handicaps, with these difficulties, but we always enjoy the sea and the view. We see a horizon (…) The ship disappears in the horizon to unknown places. We don’t see exactly where we are going but we are inside this ship and hope that it leads to a good destination towards the sun. The flowers at the beach indicate that we are also having a good time. We have experienced a lot of beautiful things, we were lying in the sun, here the chairs have become empty now, but we are still here (…). Seen from my point of view, it is a hopeful picture, which nevertheless has the shadows of everyday life, and it shows that there are also dark sides, stony paths, or you can be alone sometimes and still be together as a couple as long as it is possible. The fire is still burning, maybe a small one (…) Even our living together, our intimate life, that hasn’t been extinguished*”.

(FC26)

### 3.3. Changing as a Person during the Disease

A large number of participants explained that they perceived changing as a consequence of PD or noticing that their loved ones were no longer the same people they were before having PD. Some patients, despite noticing a change in their personality or character, were unable to put this change into words and considered their spouses better able to explain these changes. Some of the changes most frequently observed by patients or their FCs were the loss of a sense of reality and a decline in self-esteem, self-confidence, and initiative. Changes in personality including increased negativity, irritability, snappiness, selfishness, or impatience were reported by some participants:

“*I have the feeling that my personality has been turned around a bit. I am not the same person I was before. I was so friendly and nice before. And today I am almost toxic. Sometimes I also give poisonous answer to my husband. I feel like that’s not very good. But I can’t help it*” *(…)* (P28). “*And you think this change has to do with the disease?*” (R1). “*Sure, 100 per cent yes, because it has come more now*” (P28). “*Since when?*” (R1). “*Since I could no longer walk. That was the worst thing for me*”.

(P28)

Both patients and FCs frequently mentioned increased introversion and decreased talkativeness. These kind of changes in behavior, character, or personality can be very challenging for FCs:

“*That’s still a bit difficult for me now. Yes. In the past few years I had the feeling that he was somehow isolating himself and he is like in a cage with his illness. Somehow, I have the feeling that I can’t get close to him anymore as if there would be like a Parkinson’s wall between us*”.

(FC6)

“*Before the illness, she was really energetic and always had to do something, and now with the illness, I almost have to force her to go somewhere, to the theatre or the cinema or somewhere. She has all the excuses she needs not to be around many people. And yes, how should I put it, um, because of the illness she has also become more selfish. So, she comes first and then again and then maybe the others. Sometimes I’ve also said, I’m not a domiciliary care provider. You pay him and you can give him orders, but I don’t get paid*”.

(FC29)

One FC explained that her husband drastically changed during the illness due to the combination of the side effects of the oral medication and his way of coping with the disease:

“*He was so hyperactive, and I didn’t know if that was because he knew that he had a disease and he wanted to enjoy life (…) He was thinking more about himself, looking more for his own pleasure. He had no sense of time and he was looking for his pleasure. That was his first concern, to think of himself*”.

(FC2)

Other changes observed were decreased spontaneity or concentration, becoming more forgetful or hesitant to do things, or developing risky behavior (e.g., riding a motorbike without a helmet). Some participants explained that aging plays a role in changing as a person during the illness. The above-mentioned changes were considered to be rather negative by both patients and FCs. Two of the participants described some positive changes in their way of being after the PD diagnosis. For instance, becoming more patient with others or being more able to enjoy the little things in life. A couple of participants identified changes in themselves but did not consider them to be either positive or negative. For example, one explained that since he had PD, he perceived emotions differently:

“*On the one hand, you get emotional much more quickly. So, when there is an emotional situation, tears come immediately, even if I don’t want them to come. Both positively and negatively. The emotionality is actually much greater. But conversely, in the perception of happiness and unhappiness, one becomes somehow like a little more indifferent. Everything is always a little more or less good. It’s not extremely good and it’s not extremely bad either*”.

(P5)

### 3.4. DBS Improved My Life

Many participants reported that DBS greatly improved their motor skills. For instance, better body control and recovery of fine motor skills, experiencing fewer tremors, or decreased rigidity, dyskinesia, and freezing. The greater mobility from the decreased symptoms allows the patient to perform daily tasks (cooking, eating, going to bed, getting up, repairing things, walking without falling or working) more easily. This allowed them to enjoy greater autonomy and to be less afraid of doing things like going out or driving alone. Having more energy during the day, increased concentration, and being able to restart activities they were previously unable to do, such as doing sport or knitting, were also reported.

“*Since then, she can use her hand completely again. She doesn’t tremble. She can do different things by herself again. Before I had to cut the meat and everything for her, and today everything is back to normal*”.

(FC29)

**Figure 11:**
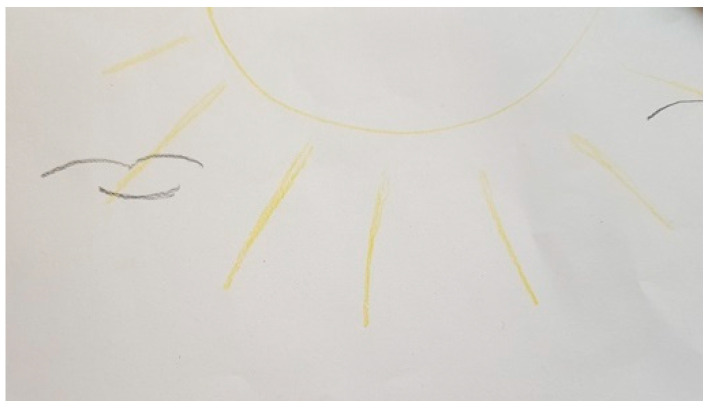
P40’s perception of DBS’s effect.

**Figure 12:**
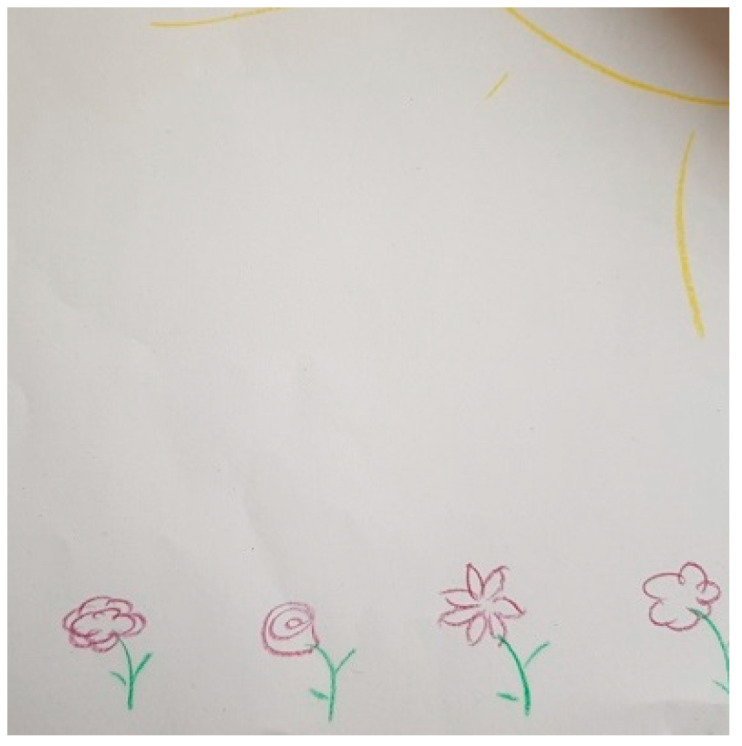
FC41’s perception of DBS’s effect.

Due to this improvement in PD symptoms, many patients and FCs described DBS as the beginning of a new life, as shown in Figure 11 and Figure 12:

“*Well, there’s a kind of rebirth, yes, with a few clouds, because it’s not easy every day. But already much, much better than before*”.

(P40)

“*And then after I drew spring, because the stimulation (DBS) is hope, renewal, and then it’s life that blooms again*”.

(FC41)

Some participants believed that their physical condition would be much worse if they had not undergone DBS:

“*If I hadn’t had the operation, I might be in a nursing home or something (…) If I hadn’t operated, I think it would probably be more difficult. Almost certainly. Anyway, my partner says that it has helped 100 per cent*”.

(P9)

“*I’m alive now and if I wouldn’t have gone through it, I’d already be underground*”.

(P21)

DBS also improved the FC’s life quality as they felt relieved by the reduction in their loved ones symptoms and were able to have more time for themselves because they were not required to be as attentive to the patient. Likewise, the patients’ motor improvement enabled them to resume hobbies with their FCs that they enjoyed doing together. The positive effects of DBS gave many participants hope and the possibility to plan for the future again:

“*Do you now feel that deep stimulation has changed your life?*” (Researcher 1). “*Yes, certainly, in the sense that I can do practically everything again. I have a perspective again, at least for the next ten years, a positive perspective. I can consciously plan things again that were previously written in the stars. I can now seriously plan them again. For example, going on a trip with my wife after retirement. We have so many plans that it makes a huge difference when you can plan again and assume that it will work out. We had all these uncertainties before. Before, I didn’t even know if I would be able to work until retirement. That is no longer a question. I’ve already agreed with my colleague that I’ll be available and able to work after retirement*”.

(P5)

In addition to the improvements in the symptoms mentioned above, the ability to speak more clearly and loudly again improved both the patient’s social life and their couple relationship.

“*He is more at ease when speaking (…) He can stay standing or go with them* [the neighbors] *to see something. Whereas before he withdrew himself a little when he had so much dyskinesia. I do think that it’s better now for the contact and the neighborhood network*”

(FC39)

“*Now it’s better after the deep brain stimulation. It is really better (…) The speaking part that is very important for me, that you can exchange and talk to each other. Of course, that became less. Parkinson’s patients also speak less, of course. That was also a huge problem for me. I like to talk about everything. I want to exchange ideas. That was no longer possible. That is better now*”.

(FC6)

**Figure 13:**
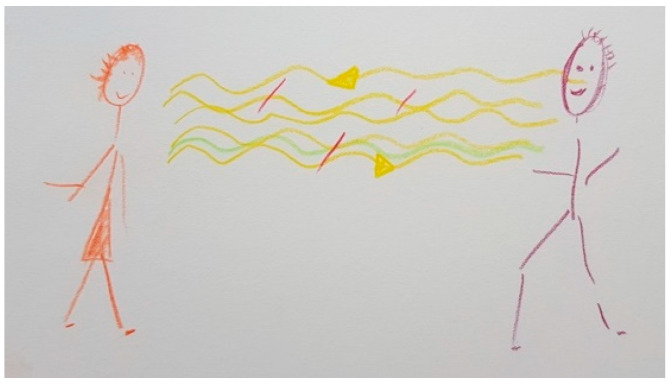
FC6’s perception of DBS’s effect.

Several FCs mentioned the recovery of facial expression as an important improvement seen with DBS. Figure 13 shows how facial expression of FC6’s husband improved in comparison with Figure 2, which led to fewer interruptions in communication and improved their couple communication. Fewer short red lines are observed in Figure 13 than in Figure 2 because of this. The question mark in Figure 2 has disappeared because she no longer needs to try as hard to understand if something is happening to her husband. The speech of her husband improved, which allowed him to express himself better, showing more empathy when speaking and communicating more fluidly.

The blue line symbolizing FC6’s efforts to communicate properly with her husband from Figure 2 is substituted in Figure 13 by a green line, which represents the improved communication and connection between FC6 and her husband. The following reason was given:

“*Now here I have the hope, partly after the deep brain stimulation, that even if it is a bit worse in between, it will get better again*”.

(FC6)

The reduction of medication and its associated side effects (such as hyperactivity, stomach problems, or lack of taste), the possibility of controlling PD symptoms without having to increase medication, and no longer experiencing the on–off effect of the medication were considered positive aspects of DBS by several participants.

“*One of my doctors described it* [DBS effects], *very well. He said that it’s like sitting in a cold room and making a fire. The fire goes up, down up, down, and you are cold and then warm, and cold and warm. And the stimulator is like you install a central heating system. So, it’s a continuous effect (…) Before* [with oral treatment], *it was high low high low, and the stimulation is straight. You have a constant effect with the stimulation*”.

(P1)

“*It doesn’t always work out equally well, but as long as it is still possible to put the power up and then it works (…) Before, it was necessary to increase the meds, and now you can do the same much easier just using electricity*”.

(P27)

A few patients mentioned the ability to modulate the amplitude of the stimulation with a remote control to better control certain symptoms as an advantage; however, most of the partients did not use it.

“*Yes, I use it occasionally, so for control, but I don’t change it every day or much. I think I’m actually not badly adjusted. What I’m going to do, maybe this week, is to lower it a bit on the other side because I’m having more and more cramps in this upper arm, which are very painful and that can help*”.

(P12)

### 3.5. I Am Treated with DBS but I Still Have Parkinson’s

Despite the improvements many patients noticed in their health, both patients and FCs highlighted that DBS is not a cure for PD. It is a therapy that can improve certain symptoms of PD but not all of them, and it does not halt the progression of the disease:

“*The disease is progressing (…) It’s going to get worse and worse*”

(P44)

“*It’s better, but you still live next to a sick person, and you sleep next to a sick person*”

(FC2)

“*I have been given a new life. Another chance, so to speak. But this chance is now increasingly limited, of course, because I realize that I can’t do many things any more (…) It’s not a cure. That’s precisely the problem (…) Other people think you are healthy again. That is a consequence of the operation, that many people in the circle of friends then thought, now you have had the operation, now you are as you were before, capable of performing, able to work under pressure. And that is simply not true (…) The effect [of DBS] was like getting a new life, but now the illness is coming back stronger*”.

(P12)

**Figure 14:**
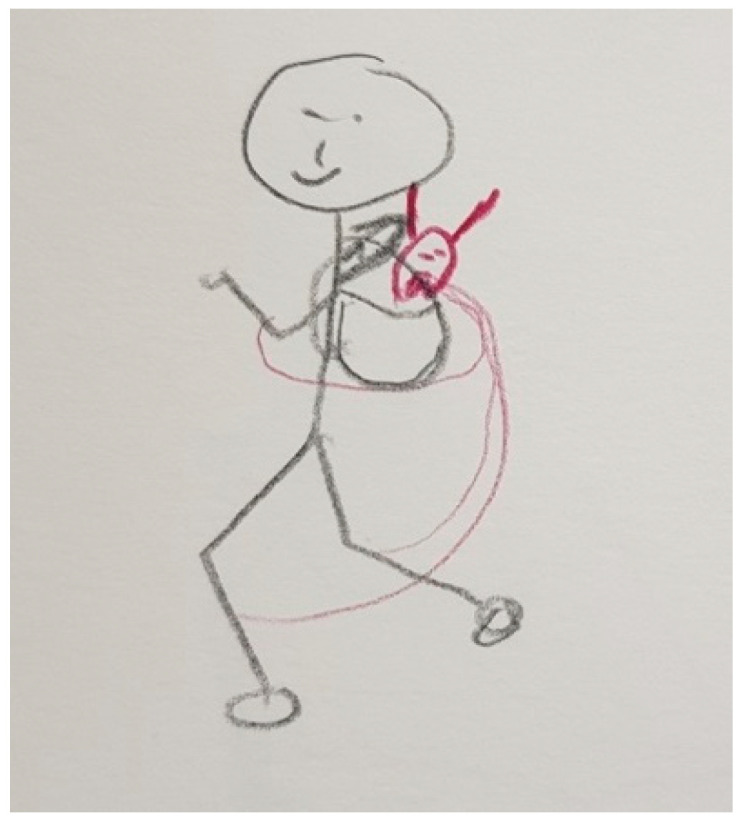
P5’s perception of DBS’s effect.

Despite knowing that the disease is still present, Figure 14 shows how some patients have the impression that they can manage the disease better with DBS than with their previous treatment.

“*I would say that the little devil is still there, but at the moment he is sitting in a backpack and is not hindering me (…) I know that [PD] is not gone. That must always be clear. Maybe it’ll get out of the backpack again and lead back again. I don’t know. The doctors think I could expect the effect of deep brain stimulation for about ten years*”.

(P5)

Although some aspects have improved greatly with DBS, others may worsen as the disease progresses. As a result, some FCs ended up taking on more and more everyday tasks.

“*It is noticeable today that everything has become a little slower (…) The asking back and forth, that has increased. In the past she cooked, I had no problem, I ate what she made. Today I have to ask her, what would you like for dinner today? That has become our daily routine, three times a day, or, in the morning, I say, what would you like, bread, everything, at noon and in the evening. Yes, that has become my task, to think a bit more for my wife as well*” (FC26). “*More after the intervention than before?*” (Researcher 1). “*Yes, before I didn’t have to think for my wife anything. She organized everything herself and was independent in every way. She managed the household, but today we have to share everything*”.

(FC26)

**Figure 15:**
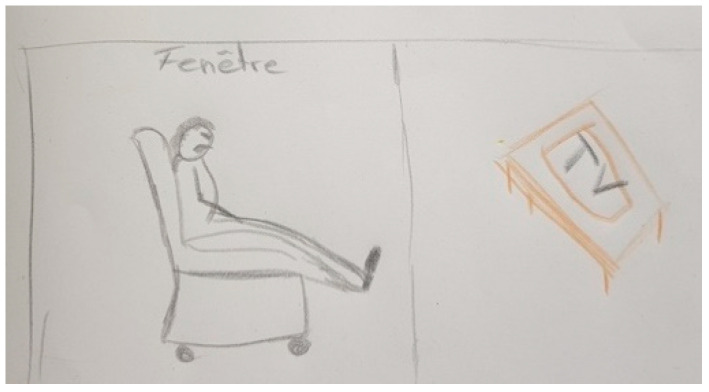
FC45’s perception of DBS’s effect.

Figure 15 shows life of her husband since he is treated with DBS because the disease started to progress quickly:

“*It simply means that life in society is different. It means that he is often in a chair at home, uh, because of fatigue. Fatigue and then walking, eh walking, it has decreased a lot too*”.

(FC45)

**Figure 16:**
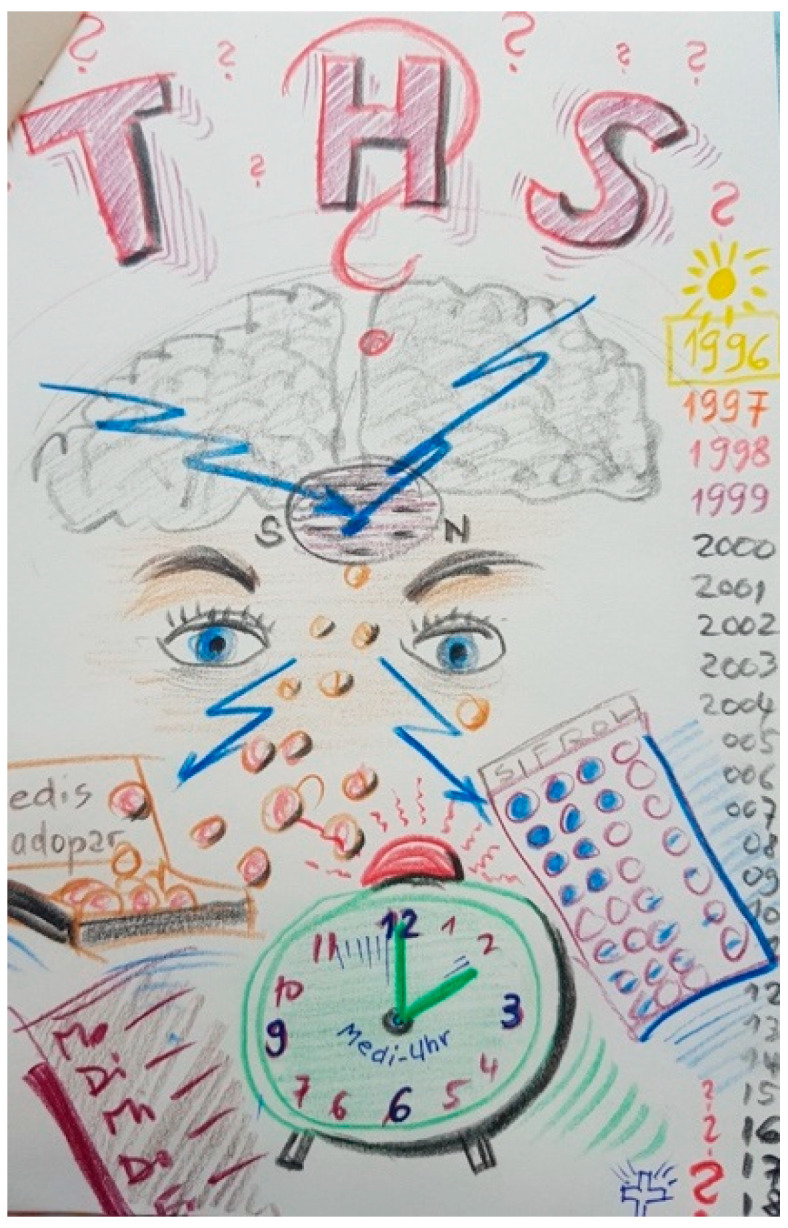
FC26’s perception of DBS’s effect.

Furthermore, being treated with DBS does not always mean the end of taking tablets; many of the participating patients had their medication dosage reduced but not stopped. Figure 16 shows how PD and the timing of medication still mark the daily routine for some patients and FCs.

“*It is a picture full of confusion. You can see the brain and the lightning that works into the substantia nigra, those are the electronic currents. And that goes out again with these daily worries, with the medication, with the appointments you have, you always have to think about it, about all kinds of things. Time runs. It’s ringing again, that’s the alarm for the tablets intake that is at the center here. Today, it controls our everyday life quite strongly (…) The eyes are perhaps a little bit empty here (…). I would say they* [the eyes] *are hopeful for the future despite all the chaos, the lightning that comes at us every day, the question marks that surround everything here, DBS, the medications, yes, and so on*”.

(FC26)

### 3.6. DBS Is Not Perfect

DBS did not work equally for all patients; many experienced great improvement from the start, while some only experienced good symptom control for a short period of time, and a minority observed little to no improvement with DBS.

“*Does the medicine work right away? Doesn’t it work yet? Uh, a lot of things are happening at the same time. It’s very difficult and each person is very different*”.

(FC45)

Although DBS contributed to improving the life quality of many of the patients, some mentioned that it is not a foolproof treatment because it can also worsen certain aspects. The worsened aspects included: their energy level, balance, speaking, coordination, or flexibility. This had an impact on the participants’ social life and in some cases led to withdrawal from certain activities or hobbies.

“*If he has to speak for a long time, usually his voice will diminish, he won’t be able to (…) He enjoys going to restaurants, eating, something he didn’t enjoy before. But on the other hand, he can’t express himself when he is in society and has to speak when there are a lot of people (…) So that’s one of the disadvantages*”.

(FC41)

It was mentioned that some aspects cannot be controlled with DBS such as fatigue, freezing episodes, or experiencing difficulty in walking:

“*Walking is going down, but the doctor surgeon, neurologist said that deep stimulation does nothing for walking or very little. We had the impression that it was very difficult to adjust it, to make a fine adjustment*”.

(P39)

A few participants mentioned being somewhat disappointed with the treatment because they expected it to eliminate all symptoms permanently, and only partial improvement was achieved:

“*I have to say, I expected more. I thought after the brain operation everything would be fine, yes, fine, the hope was there, now everything will be fine again. If I am stimulated every day afterwards it’ll be like before. But that wasn’t the case. That was only at the beginning. The shared joy (…) It’s only possible to adjust it so that it is optimal (…) And that ideal point, was not always ideal. At the beginning, they had to change it a bit up, a bit down, and then it is found wasn’t the best result. Ah, it’s the best possible, but not what we had hoped for*”.

(FC26)

One patient mentioned that her husband hoped to be able to travel more with her after the improvements achieved with DBS, whereas she did not feel able to:

“*Yes, well my husband, he would rather go on holiday even more than I would. For me it’s always in a new place is already a bit stressful. So I notice that. I told you, I’m not so resilient anymore. So packing, that’s hard for me*”.

(P35)

**Figure 17:**
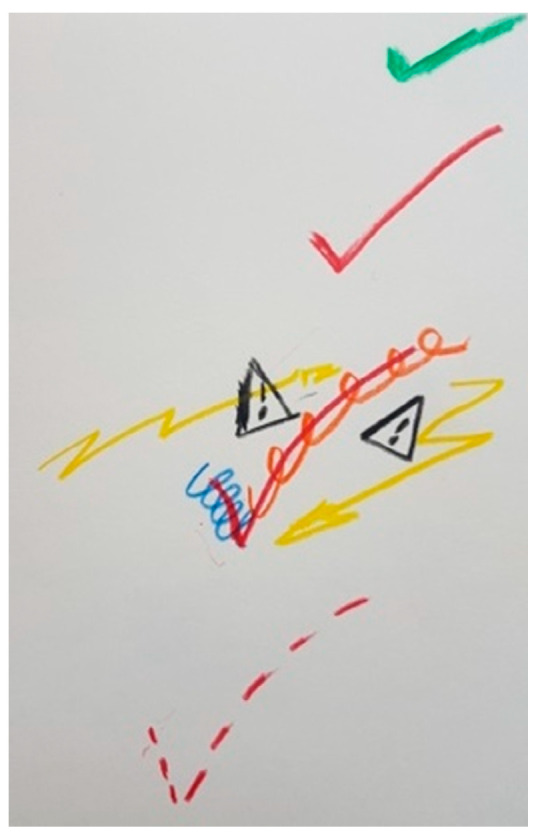
P1’s perception of DBS effects.

Figure 17 shows some of the negative experiences like the occurrence of infection due to the surgery or depression as a side effect of DBS, which some patients went through, even if they were satisfied with the treatment.

“*This is at the beginning, and there is no signal, or few signals before the DBS is implanted, and afterwards there is a positive V. It’s a “big turn” but it is still fragile, and this is the infection [the triangles with the exclamation mark]. After the infection, it went well, but now, I have to say I always have the impression that in the depression, the stimulator is a very important element*”.

(P1)

### 3.7. Being Different after DBS

A few participants described changes in their personality or their loved ones since the treatment with DBS started. The following changes were reported: impulsive behavior, irritability, excessive euphoria, or becoming more selfish, emotional, impatient, or withdrawn:

“*He takes care of things he likes and doesn’t need to have a lot of people around him*” (FC45). “*And that was also like that before the operation?*” (Researcher 2). “*There was already a little bit like that before the operation, but it was really minimal. It didn’t happen daily or anything like that. (…) And then [after DBS intervention], he became much more sensitive. For example, if you watch a film and there are emotions involved in that film, he would cry straight away. You can see the tears. He was never like that*”.

(FC45)

“*I was irritable, belligerent, and freaked out. I drove everyone crazy in the hospital*” (P23). “*And this was immediately after the operation?*” (Researcher 1). “*Immediately? Yes. Maybe three weeks later as well*” (P23). “*And now do you still feel different?*” (Researcher 1). “*Yeah. Still not good*”.

(P21)

Some of the described changes occurred in the first weeks or months after the surgery and disappeared after a few months, while in other cases, these changes in personality or behavior remained. In some cases, these changes were accentuations of personality traits that had developed during the course of disease. Sudden mood changes and manic or depressive episodes never experienced before DBS were also described by a few participants:

“*He really had a personality change for a short period of time and also a manic phase. He was completely different for a while*” (FC37). “*What do you mean by manic phase?*” (Researcher 1). “*Yes, after the operation he was really changed in his manner, that he for example/that he complimented me or hugged me when greeting me, as he never did before (…) He bought an expensive watch and booked holidays, big holidays, without discussing it with my mother. And also wanted to write a book. Yes, things like that*”.

(FC37)

Mood swings brought stress into the couple life of some participants:

“*After DBS, she had a bit of trouble when I had so many ups and I made a lot of quick decisions. I invited people to our place and so on, and she didn’t appreciate that so much. Just because she also didn’t like it so much when it went down again after DBS. It wasn’t depression, but depressive moods*”.

(P27)

One of the FCs explained that they were not prepared for the occurrence of depressive episodes with DBS because they were not informed about it:

“*And he had it again a fortnight ago. So, depression-like episodes (…) No one told me that could happen (…) He wasn’t told either. We talked about it recently. I told him, why don’t you ask that in the next consultation? I told him that when he has an examination in hospital, then he should ask whether this side effect is possible*”.

(FC17)

One patient explained that being treated with DBS made her worry about things she didn’t worry about before and that this led to arguments with her partner that she did not have before:

“*My partner said, oh, you have to get an induction cooker. And then I immediately thought, magnetic fields. And then it was when I first googled that and it’s a topic in the media and that’s why I thought, I don’t want it. I don’t want that in ten years will be found out that it’s harmful for the brain or the battery. And those are moments that are different with my partner*”.

(P35)

Some perceived changes, such as finding themselves or their loved ones more relaxed, positive, disinhibited, or with a cheekier sense of humor were positively valued by a few participants. One patient explained that it was positive for him to be more open to talk to people he did not know, including strangers. A couple of patients explained that since they were treated with DBS, they felt like the person they were before the disease, while others described feeling like a new person because they have a new life. Some FCs also reported the impression that their loved ones resembled the people they were before the disease again or the feeling that both of them were given a new life after the improvement of PD symptoms due to DBS. One of the patients described not feeling like the same person as before DBS intervention differently to experiencing changes in personality or feeling like a new person:

“*I don’t have the feeling that I’ve become a different person. I haven’t but at the same time I’m not the same. I’m much more anxious. (…) It [DBS intervention] was certainly a borderline experience. It’s a borderline experience like a birth. I also compare it to a new life that I got. Other people don’t have this borderline experience, and that creates often a distance to others*”.

(P12)

The presence of a device in the brain was not described as an element changing them as people, but as something that is now part of their lives. Although some of them could notice the device being inside their, or their partners, bodies, it was not a problem for them:

“*I don’t have the feeling that there is something in there. I do notice it, of course it is a foreign body in my body, but I live with it now and not badly*”.

(P7)

“*No, nothing bothers me about her, even that she has such a device above her chest that you can see and feel, that doesn’t bother me. (…) That belongs to my wife. Exactly. It’s not a foreign body from my point of view. I don’t perceive her as my wife, who has electronics in her brain. I just don’t think about it at all*”.

(FC26)

One of the patients called himself a cyborg, explaining that this term did not have a negative connotation for him, although it did for other people:

“*I’ve always said I’m a so-called cyborg now. The funny thing was that I said this once to my neurologist that I would be a cyborg afterwards. Then he immediately objected and said, no, no, you are still a human being*”.

(P5)

## 4. Discussion

Our study examines the perception of patients and FCs of PD and DBS and the changes in life that they face as a consequence. We observed great heterogeneity in PD symptoms, their progression, the effects of DBS, and the perception of patients and FCs of PD as a disease and DBS as a therapy. Therefore, “everyone’s Parkinson is different” is the overarching theme. Each person has a different experience of the disease and reacts differently to it. This means that PD impacts their daily lives and relationships differently and that their experience with the treatment is different. Therefore, different coping strategies were described by patients and FCs. Our results show that what works for some does not work for others. However, there is one strategy that helps most patients cope with PD symptoms and that FCs recommend. This strategy involves patients doing physical activity or concentrating on something they enjoy, because they experience fewer symptoms while doing it. This information could be relevant for clinicians when they provide patients and FCs with strategies to cope with their symptoms in day-to-day life.

We decided to implement a multimodal approach with the aim of capturing a more detailed account of the participants’ experiences [48]. Complementing the interviews with drawing gives participants the opportunity to convey their emotions through the use of color and shape, giving their words a new dimension. This provides the researcher with additional insights into their experiences [59,65,66]. The drawings allowed us to identify major difficulties and concerns that participants had in relation to PD, as well as the implications of DBS as a therapy on their lives. Drawing gives the participants in qualitative studies a tool to reveal feelings and aspects of their internal world that are not always visible [67]. For instance, in Figure 2, FC6 uses lines and curves of different colors and shapes to visually describe the impact of PD on her communication with her husband and the internal struggle she felt due the breaking of the emotional connection between her and her husband. The short lines in red that we saw earlier represented both the interruptions in communication and the emotional pain caused by this situation. This is similar to the representations of physical pain drawn by some participants in a study on the pictorial representation of chronic pain [66]. In this study, two participants represent the process of managing pain with blue as FC6 does when she draws a blue line to symbolize her efforts on maintaining the connection and communication with her husband.

Another study on chronic pain shows the how the participants associate pain with certain figures, in the same way that some of our participants did when they drew how they visually perceived PD [68]. For example, Figure 3 and Figure 5 illustrate the feeling of PD being a constant presence in their lives either in the form of a shadow behind them or a devil pulling them along. Figure 4 represents the duality of PD as a demon and a fairy, showing the loss of control for both the patient and FC due to PD. In this drawing, normality is associated with the absence of symptoms, represented by the fairy, which allows the participants to carry out their daily routine without interruptions. However, this sense of normality disappears when the symptoms associated with the demon manifest themselves, which cannot always be foreseen or anticipated. Thus, Figure 4 shows us the unpredictability and uncertainty that characterize PD and how this is also a burden for the relatives.

In Figure 8 and Figure 9, we see different symbolism for PD: the grim reaper who takes away the freedom of the person or the glue that does not allow the patient to lift their feet from the ground. They both represent the lack of freedom that PD enforces on the life of patients and FCs, which marks a turning point in the life of both patients and FCs. Therefore, DBS also marked a before and after point for those who had a good response to the treatment, which is represented through Figure 6, Figure 7, Figure 11 and Figure 12. On the one hand, Figure 6 and Figure 7 show the disease in the form of a storm and the autumn leaves denoting the end of summer. On the other hand, Figure 11 and Figure 12 show the association between life with DBS and the resurgence of a sunny day or the blossoming of flowers in spring. Other studies also show how images of seasonal or weather change represent life transition for people with different health conditions [59]. In the case of our participants, the treatment with DBS implied a new period of life that brings better quality of life and a sense of greater control, as we can also see in Figure 14, where the devil is on the patient’s back and no longer behind him pulling him back. However, this devil could get off and start holding the patient back again, which denotes the patients’ concern about the evolution of PD and the awareness of PD not being a cure. Furthermore, not all participants benefited equally from the therapeutic effect of DBS. This is reflected in Figure 15, showing life with DBS when it does not have the expected effect or when the effect disappears due to the progression of the disease.

All these drawings, and the explanations that accompany them, show us the complexity of living with PD and receiving treatment with DBS. It is a shared experience between patients and their FCs, as Figure 10 and Figure 16 show us. In these images, FC26 shows us how they go through PD and DBS together, which has been also described by other authors [69]. The FCs (especially in the case of spouses) are not only emotionally involved in the illness of their loved ones, but they also provide care at home. Not only do they need to learn to manage the internal impact on their lives that the fact that their spouses have a chronic illness has, in most cases, they also adapt their routines to assist their loved ones in different tasks, such as helping with personal care and hygiene, managing the patient’s treatment, or organizing medical appointments [50,52,69]. In most cases, they are a great source of support and understanding for patients [49,70]. In addition, certain symptoms of PD disease (e.g., slowness of movement, fatigue, or psychiatric manifestations) or some side-effects of dopaminergic medication (e.g., hyperactivity or hypersexuality) or DBS (e.g., temporary mania or mood changes) add great pressure on the shoulders of FCs. Previous work highlights the need to consider the impact of PD on FCs’ wellbeing and their need to receive support from health services to deal with the situation [70,71,72]. Self-help groups are an important source of support for some FCs because they allow them to find information about PD and DBS and emotional support by sharing experiences with other people in the same situation [73]. This is especially important because the burden of the disease on relatives does not disappear with DBS; nonetheless, it is mitigated for some time when DBS goes well [69,74]. Thus, it is important to help FCs, in addition to patients, to understand the implications and limitations of DBS in order to prevent unrealistic expectations (e.g., expecting life to be the same as before the disease) [75,76]. Both patients and FCs should also be prepared for the fact that DBS may not resolve certain PD symptoms or may cause side effects, affecting patients’ personality or behavior [76].

We analyzed the patients’ process of change during the disease and the treatment from their own perspective and that of their FCs. In order to assess the impact of both PD and DBS on patients’ identity, personality, or behavior, we asked both patients and FCs whether they had the feeling that they or their loved ones had changed as people. This decision was made because the concepts of personality or identity do not have a universal meaning and depend on culture and individual evaluations [77]. We therefore consider that the idea of changing as a person could be understood in a more homogeneous way. While some participants did mention the concepts of personality or identity directly when answering this question, not all of them did. In the bioethics literature, it is common that the side effects of DBS are portrayed as a threat to patients’ personality, identity, agency, and self-perception [32,78,79,80]. Although many of these articles are not based on firsthand studies, there are studies showing some post-operative changes that could negatively affect personality, behavior, or mood (e.g., impulsive behavior, depression, mania) [35,81,82,83,84,85]. While these may occur, it should not be forgotten that PD often presents with psychological and psychiatric manifestations (e.g., depression, anxiety, hallucination, apathy) [86] and dopaminergic medication like dopamine agonists can also lead to impulsive behavior such as compulsive buying, hyperactivity, or sexual behavior [87,88,89,90]. For instance, some of our participants described changes in their personality prior to undergoing the DBS intervention such as hypersexuality, compulsive shopping, or sudden changes in mood, which in the bioethics literature are normally associated with DBS. Given that many patients continue to require oral medication (at lower doses) and the disease keeps progressing, it is difficult for clinicians, researchers, and also patients to discriminate between PD symptoms and drug- or DBS-induced side effects. Examples of such difficulty may be the mention of anosmia as a side effect of the medication by the participants, although in fact it is an early symptom of PD, or the consideration of dyskinesia as a PD symptom, when it is a side effect of prolonged treatment with levodopa [91,92].

Furthermore, our study reveals that the patients’ experiences with DBS are inherently entangled with their experience of suffering from PD, which is still present. Therefore, the whole experience of both suffering from a chronic disease and being treated for it has an impact on the patients’ narrative and not just the fact of being treated with DBS, as is often portrayed in the literature on neuroethics [30,79,80,93]. Thus, patients and FCs have to integrate all these elements and changes, which requires a process of adaptation and adjustment that every person experiences in a different way. Whilst we did not identify severe problems with social adjustment as other authors did [31,46], we did observe that our participants went through a process of adaptation and adjustment that every person experienced in a different way. However, in general, patients with positive therapeutic results with DBS showed satisfaction with their life and the improvements in their symptoms.

Some authors address the issue of the burden of normality by describing it as the reconceptualization of the patient’s identity from chronically ill to “cured” due to the disappearance or improvement of PD symptoms as a result of DBS [37,94,95,96,97,98,99]. According to our data, the patients who participated in our study did not consider themselves cured because they had fewer or no symptoms of the disease or as if they had lost the “disease label” [100]. All of them, including those with very positive experiences with the device, were aware that the disease was still there and could worsen again over time. This awareness of the presence of the disease is evident from Figure 15, in which the devil-shaped PD is under control in the backpack but has not disappeared and may come down to the ground again. Furthermore, some debilitating PD symptoms such as fatigue, freezing of gait, and balance impairment could not be targeted by DBS [76]. The closest experience we found to the burden of normality but without actually being so was the description of a patient of DBS as a “borderline experience” for two reasons: the experience of the surgery and the fact that she was given a new life that allowed her to do many things she could not do before. Although the reduction in symptoms was something she enjoyed, she reported feeling certain social distance from people who had not experienced undergoing DBS. Nevertheless, the improvement in PD symptoms did not led her to experience radical adjustment problems or behavioral changes negatively affecting her as shown in other studies [50,52,69].

In the bioethics literature, it has also been described how couples can find themselves under pressure following symptom improvement and relief from withdrawal or reduction of medication [32,97,100]. However, we have rather observed an improvement in spousal relationships as a result of motor improvement. What was a source of stress for a few couples were some of the side effects of DBS such as impulsivity or depressive episodes, which usually occur in the first few months with DBS and are often resolved by adjusting the stimulation parameters [46,100,101,102]. Nevertheless, PD symptoms, both motor and non-motor, as well as drug-induced side effects, pose important challenges for the couple’s relationships. Nevertheless, PD symptoms, both motor and non-motor, as well as the side-effects of dopamine agonists, may be even more challenging for the couple’s relationships [89,90,103]. In this regard, a number of participants reported a series of difficult situations they had experienced: the progressive change of the spouse’s role from partner to caregiver, the lack of communication of some couples about the disease, the impossibility of sharing some hobbies or interests, the differences of opinion on whether or not to talk about the disease, the pressure on the partner dealing with episodes of addiction or hyperactivity caused by dopaminergic medication, and the marital crises, which in one case ended in divorce. Therefore, we consider that in the case of our participants, DBS improved their relationships due to the reduction of PD symptoms and lower medication, which decreased the side effects associated with it.

Based on our results, we do not share the idea defended by other authors that patients undergo DBS with the objective of changing or enhancing their personality or their way of being (at least not in the case of PD) [79,93,104,105]. Although some patients since being treated with DBS did experience some changes in their personality, behavior, or mood (e.g., being more positive, relaxed, or disinhibited) that were welcomed by them, this was not the aim of the therapy. It is also important to remark that different individuals may value the same side effect either as positive or negative depending on their character, life circumstances, or life narrative [39,106]. Our participants did not use the remote control with the aim of changing or stimulating their personality, as described in the literature in bioethics [104], but rather to better control or relieve certain motor symptoms.

Another aspect that has received much attention in the literature is the relationship between patients and the DBS device. As a device partially implanted in the brain and considering the close relationship between brain and mind, some authors have underlined the importance of carrying out an assessment of the psychosocial consequences of this treatment [107]. Other authors consider that DBS may induce self-estrangement as some patients struggle finding themselves after surgery [108]. It has been described that some patients had the feeling as if they had lost their true self or as if they would be a machine or a cyborg [31,32,46,95,109]. In contrast, our participants did not find it problematic having a device in their brain or their loved ones having it. Even one of our participants defined himself as a cyborg without feeling lost or alienated. He was therefore surprised by the reaction of his doctor, who emphasized that he was not a cyborg but a person. We believe that DBS (especially if it works well) may become a constitutive dimension of lived experience, which does not need to cause self-alienation in the person [109]. In fact, DBS can also have a restorative effect on the person [106]. Our participants had rather the feeling that they have become more themselves because they see themselves as more identified with the person they were before the disease. Others felt they had received a new life that could enjoy more than the one they had before the surgery, which is described in the medical literature as a “second honeymoon” [110]. The patient who described having undergone DBS as a “borderline experience” in her life described how she did not feel the same anymore after DBS without having become a different person. However, this feeling was not due to the fact that she had a device inside her brain but due to the experience of having undergone invasive surgery and having been given a new life. We believe that the embodiment of the device as a part of the patient’s body may be the reason why our patients did not experience self-estrangement, even though they may have noticed changes in themselves during the time they have been treated with DBS.

In view of all the above, we did not observe a deteriorative post-DBS biographical disruption as other authors described [31,95]; nonetheless, we did observe a post-PD biographical disruption [111]. PD as chronic disease alters the structures of everyday life and affects self-perception, modifying patients’ sense of self and agency [112,113]. It also challenges the interrelationship between mind and body because the body does not always act when and how the mind asks it to act [114,115]. Furthermore, the diagnosis of PD and its progression implies for both patients and FCs a continuous reinterpretation of the past, the present, and the future, and DBS is part of this reinterpretation but not the only cause [116].

### Strengths, Limitations, and Future Directions

Our study applies the narrative DIPEx approach to explore how patients and FCs perceive and experience PD and DBS, which gives the participants a greater control over the structure, length, and content of the interview [57]. This facilitated long and in-depth discussions with the participants, which led to rich and credible results due to the strategies of prolonged engagement and persistent observation [117]. Furthermore, most participants, both patients and FCs, experienced a beneficial emotional effect from having been listened to and from their personal stories having been taken into account for research [52]. We consider that this feeling of being valued and trusted by us during the interview encouraged them to talk very freely about their emotions, fears, and needs. Another strength has been the inclusion of FCs in the study, which allowed us to examine their perception of PD and DBS and their role in supporting the management of the disease and the treatment at home. The account of FCs also served to complement the information given by the patients about their own experiences, especially in cases where the disease was more advanced, and hence, their inclusion provided us with valuable information that highly enriched our study. For data analysis, we have employed a hybrid process of inductive and deductive thematic analysis. This method has been adequate to categorize and analyze in depth a large number of experiences and to explore the differences between the patient’s perspective and the concerns shown in the bioethics literature.

Furthermore, we consider our multimodal approach as a major strength of our study, which is the first one that has been conducted to explore the experiences of patients and FCs with both PD and DBS. Drawing has been explored as a possible tool for early diagnosis of PD but not as a qualitative method to analyze experiences with PD [118,119]. We believe that the collection of drawings as a complement to the interviews provided us with additional insights into the participants’ internal world and their subjective experiences with PD and DBS. It offered us access to nonverbal meanings and to a qualitatively different aspect of the participants’ experiences [53,120]. Furthermore, the action of drawing allowed our participants to reflect on their own narrative and visually show the impact of PD and DBS [121,122,123].

For all these reasons, we can argue that our study applied rigorous qualitative methodology in data collection and analysis that meet the criteria of credibility, transferability, dependability, confirmability, and reflexivity, ensuring the trustworthiness of our results [117].

We identified five possible limitations in our study due to its design and methodology:

- Firstly, our study was very comprehensive and did not focus specifically on one issue in relation to PD or DBS as other studies did. Therefore, some issues may have been missed during the interviews, such as the burden of normality, particularly in FCs. This particular topic should be investigated in more detail in the future as it is a very underrepresented topic in the medical literature.
- The participants were interviewed only after being treated with DBS and not before they started receiving this treatment. Although all participants were asked questions about their daily lives and how they were doing before treatment, those who have lived with PD and DBS for a longer period of time may have lost perspective on the before and after. This may have led to recall bias.
- Some patients who initially showed interest in the study finally decided not to participate because they were going through a difficult time with DBS side effects. Therefore, we missed some negative experiences with DBS due to the fact that people who have bad experiences are often more reluctant to share their experiences than those who have had positive experiences with the treatment.
- PD patients treated with DBS are a defined sub-cohort of PD and are not representative of the entire PD population. For example, patients within the first years after the first diagnosis (i.e., <5 years) are not represented, since DBS is usually not provided at this stage of the disease. Furthermore, patients experiencing moderate to severe dementia or with lack of physical or psychological resilience were excluded from the study. This means that the population of patients at a very advanced stage of the disease is not represented in our study either. We are aware that this exclusion may have led to inclusion bias. However, we could not include participants unable to hold a long conversation sharing stories over different time frames (e.g., before and after DBS), which is very difficult for patients with advanced PD. Therefore, we consider that despite the risk of inclusion bias, our study applied the best sampling strategy for the objectives of our study and its methodology.
- Only patients treated from 6 months to 10 years with DBS were included to have a broad spectrum of experiences with DBS at different stages. Further studies are needed to delve into the individual patients’ difficulties and needs at each stage of the treatment. Other issues need further elucidation, such as patient and FC experiences with the side-effects resulting from dopaminergic treatment or the impact of memories of DBS surgery.

## 5. Conclusions

This study applied a multimodal approach through narrative semi-structured interviews and drawings to analyze the experiences of nineteen patients and seventeen FCs with PD and DBS. We explored the heterogeneity that defines PD, which is visible in both the manifestation of the disease and in the way of coping with it, as both aspects change from person to person. Although it does not affect everyone in the same way, our results show the great impact of PD on different aspects of daily life, including self-care, housework, hobbies, work, self-perception, plans for the future, and relationships with partners, family, and friends. Moreover, it does not only affect patients but also their FCs, who have to cope with a change in their role within the couple and/or the family and restructure their daily life to adapt to the patients’ needs.

Our findings show how DBS, without being perceived as a cure for all PD symptoms or its progression, is a treatment that in many cases improves the motor skills of patients. This improvement translates into greater autonomy and a better quality of life for both patients and their families. However, in some cases, the desired therapeutic effect of DBS was not achieved or disappeared over time due to disease progression. Whilst possible DBS side effects may have an impact on the patients’ personality and behavior, PD symptoms and dopaminergic medication side effects also have a great impact on personality and self-perception. Nevertheless, these aspects are less mentioned in the bioethics literature. Another aspect that deserves further investigation is the burden of normality not only in patients but also in FCs, as with regard to FCs, this topic is underrepresented in the literature. We suggest the use of multimodal research approaches to explore these aspects because it gives participants the opportunity both to convey emotions through the use of color and shape and to visually share their greatest struggles and concerns with the disease and the treatment. In this way, researchers will have access to valuable additional information on under-studied topics, which will allow healthcare professionals to better understand the specific concerns and needs of their patients with PD and their FCs. Our findings may moreover support clinicians in better informing patients and FCs about PD symptoms or DBS side effects in a way that is more focused on their needs, priorities, and fears. In addition, we believe that asking patients to draw can be a useful tool for clinicians when addressing sensitive topics during consultation to better understand the perspective of patients.

## Figures and Tables

**Table 1:** Description of DBS patients and FCs. ^a^ Averages are given in the order range, mean, and median.

Group	Average Age at Diagnosis ^a^	Number	Average Age at Interview ^a^	Gender (F, M)	Average Years with DBS ^a^
Patients	37–71, 50.4, 50	19	54–75, 67.2, 71	6, 13	1–10, 4.7, 2
FCs	21–65, 48.7, 48	17	30–88, 64.2, 64	13, 4	-

**Table 2:** Descriptive themes.

**1. Everyone’s Parkinson is different**	*1.1. Different symptoms and disease duration* *1.2. Different perception of the disease* *1.3. Different reaction to drugs* *1.4. Different difficulties and changes that led to different daily routines* *1.5. Different impact on the family and couple relationships and on social life* *1.6. Different coping strategies*
**2. Changing through PD together**	
**3. Changing as a person during the disease**	
**4. DBS improved my life**	
**5. I am treated with DBS, but I still have PD**	
**6. DBS is not perfect**	
**7. Being different after DBS**	

## Data Availability

Some of the data presented in this study will be made public in 2022 on the Website www.dipex.ch (accessed on 4 May 2021).
